# PRIMPOL ensures robust handoff between on-the-fly and post-replicative DNA lesion bypass

**DOI:** 10.1093/nar/gkad1054

**Published:** 2023-11-16

**Authors:** Christopher Mellor, Joelle Nassar, Saša Šviković, Julian E Sale

**Affiliations:** Division of Protein & Nucleic Acid Chemistry, Medical Research Council Laboratory of Molecular Biology, Francis Crick Avenue, Cambridge CB2 0QH, UK; Division of Protein & Nucleic Acid Chemistry, Medical Research Council Laboratory of Molecular Biology, Francis Crick Avenue, Cambridge CB2 0QH, UK; Division of Protein & Nucleic Acid Chemistry, Medical Research Council Laboratory of Molecular Biology, Francis Crick Avenue, Cambridge CB2 0QH, UK; Division of Protein & Nucleic Acid Chemistry, Medical Research Council Laboratory of Molecular Biology, Francis Crick Avenue, Cambridge CB2 0QH, UK

## Abstract

The primase/polymerase PRIMPOL restarts DNA synthesis when replication is arrested by template impediments. However, we do not have a comprehensive view of how PRIMPOL-dependent repriming integrates with the main pathways of damage tolerance, REV1-dependent ‘on-the-fly’ lesion bypass at the fork and PCNA ubiquitination-dependent post-replicative gap filling. Guided by genome-wide CRISPR/Cas9 screens to survey the genetic interactions of PRIMPOL in a non-transformed and p53-proficient human cell line, we find that PRIMPOL is needed for cell survival following loss of the Y-family polymerases REV1 and POLη in a lesion-dependent manner, while it plays a broader role in promoting survival of cells lacking PCNA K164-dependent post-replicative gap filling. Thus, while REV1- and PCNA K164R-bypass provide two layers of protection to ensure effective damage tolerance, PRIMPOL is required to maximise the effectiveness of the interaction between them. We propose this is through the restriction of post-replicative gap length provided by PRIMPOL-dependent repriming.

## Graphical Abstract

**Graphical Abstract:**
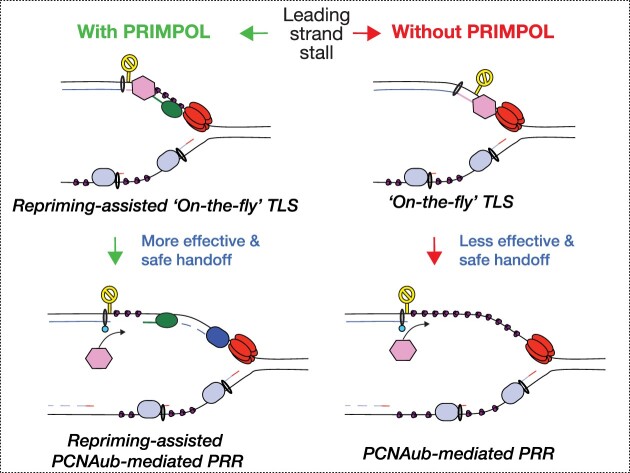

## Introduction

DNA replication impediments, including DNA lesions induced by DNA damaging agents, can lead to stalling and uncoupling of the replication fork (1,2), which if unresolved, may trigger fork collapse (reviewed in (3)). Cells have therefore evolved multiple DNA damage tolerance pathways which act to resolve stalled DNA synthesis. Classical models of DNA damage tolerance centre on two processes—error-prone translesion synthesis (TLS) and error-free template switching (4–6).

In higher eukaryotes, DNA damage tolerance by TLS utilises five specialised DNA polymerases, POLκ, POLη, POLι and REV1 from the Y-family and POLζ from the B-family. The structural features of each polymerase endows them with differing abilities to bypass specific lesions. However, their use is characterised by increased mutagenesis compared to the core replisome because DNA lesions are frequently non- or mis-instructional and the polymerases themselves are intrinsically error-prone and lack proof-reading (reviewed in (7,5)). Models of eukaryotic TLS regulation have focussed on the monoubiquitination of PCNA at Lys164 (K164) by the ubiquitin E2 conjugating enzyme UBE2A and E3 ubiquitin ligase RAD18, which is stimulated by RPA-coated ssDNA (8–11). Monoubiquitination of PCNA stimulates recruitment of TLS polymerases to a stalled replication fork through their C-terminal PCNA-binding and ubiquitin-binding domains (reviewed in (7)). However, PCNA monoubiquitination is not essential for TLS polymerase function or TLS-mediated mutagenesis, as demonstrated in both *in vitro* reconstituted and cellular systems (12–17). Following UV-C irradiation of chicken DT40 cells, PCNA K164 ubiquitination is required for filling post-replicative gaps but is not required to maintain fork progression, which instead requires the C-terminus of REV1 (15), which coordinates TLS by interacting with PCNA and the other TLS polymerases (7,18–21). Thus, there are temporally distinct pathways of TLS with distinct genetic requirements: ‘on-the-fly’ bypass (15) is independent of PCNA monoubiquitination, but dependent on REV1, while post-replicative lesion bypass and gap filling is dependent on PCNA ubiquitination.

PCNA ubiquitination not only promotes TLS but is also linked to template switching, which refers to the use of an undamaged DNA template as an alternative substrate for DNA replication, and therefore generally considered an error-free mode of DNA damage tolerance (reviewed in (4,5,22)). Strand invasion into the newly synthesised undamaged sister chromatid, similar to classical HR models, represents the simplest model for template switching (23,24). An alternate model is replication fork reversal, where the stalled replication fork is converted from a three-stranded to a four-stranded structure through annealing of the newly synthesised DNA strands (25). This four-stranded ‘chicken foot’ structure returns the DNA lesion to double-stranded DNA (dsDNA), stabilises the fork and ultimately allows bypass and/or repair of the lesion (26,27). Recent studies have provided evidence that fork reversal occurs in eukaryotes in response to a wide range of genotoxic agents (28,29). Key enzymes mediating the process have been uncovered including the PCNA K164 E3 ligases HLTF and SHPRH (30–32) and the SWI/SNF2 family members SMARCAL1 and ZRANB3, which are involved in fork remodelling and reversal (33–36).

The existence of the human *PRIMPOL* gene was first predicted in 2005 through the *in silico* classification of members of the archaeo-eukaryotic primase (AEP) superfamily (37). Subsequent genetic and biochemical characterisation identified PRIMPOL as a protein possessing activities both as a primase and as a TLS polymerase (38–41). Restart of synthesis past DNA replication impediments had long been considered as a potential mechanism for the tolerance of replication impediments (1). However, recent biochemical studies of the reconstituted yeast replisome show that leading strand repriming by POLα-primase is very inefficient (42,43). The structural explanation for this is that the leading strand emerges from the CMG helicase a considerable distance from the priming active site of POLα-primase, an arrangement conserved between the yeast and human replisomes (44). Consequently, the identification of a second eukaryotic primase with a potential role in DNA damage tolerance was significant.

Exactly how PRIMPOL-mediated repriming fits into existing models of DNA damage tolerance remains to be clarified. Current evidence supports the primase activity of PRIMPOL as being the primary contributor to DNA damage tolerance rather than its ability to perform TLS (39,45–47). As well as repriming after classical base damage, PRIMPOL also reprimes after DNA secondary structures (48,49), chain terminating nucleosides (46) and facilitates replication traverse of interstrand crosslinks (50). Several recent studies have shown how the ssDNA gaps generated by PRIMPOL-mediated repriming are filled both through further resection followed by homologous recombination (HR) and through post-replicative TLS mediated by REV1/POLζ (51–53). Situations that increase PRIMPOL deployment have also been uncovered. Impairing fork reversal, through depletion of HLTF or SMARCAL1, leads to increased use of PRIMPOL-mediated repriming (36,47,52). In addition, the cellular sensitivity to DNA damaging agents following loss of TLS, exemplified by POLη in human cells or both POLη and POLζ in avian DT40 cells, is modestly increased by concomitant loss of PRIMPOL (41,54,55). The non-epistatic nature of POLη and PRIMPOL is linked with an increased deficity in fork progression on damaged templates, likely reflecting the interaction of PRIMPOL with on-the-fly deployment of TLS (41,54,55), distinct from the post-replicative gap-filling TLS by REV1/POLζ following PRIMPOL deployment (52,53).

Curiously, despite its apparently key role in DNA damage tolerance, loss of PRIMPOL alone does not generally induce sensitivity to DNA damaging agents in human cells (54). In addition, loss of PRIMPOL results in little overt phenotypic consequence, although there is a notable dependence on PRIMPOL in the haematopoetic stem cell compartment upon stimulation of proliferation through stress (56). In this work, we aimed to place PRIMPOL in its genetic context in non-cancerous human cells through a systematic study of its interactions with DNA damage tolerance pathways. To do this, we initially employed genome-wide CRISPR/Cas9 knockout (KO) screens to guide the creation of isogenic single and combination mutant cell lines in the human TK6 cell line. We find that PRIMPOL loss enhances the DNA damage sensitivity of cells lacking either on-the-fly translesion synthesis or PCNA K164-dependent post-replicative damage bypass, suggesting that PRIMPOL-dependent repriming facilitates the effective collaboration of these temporally distinct layers of damage tolerance.

## Materials and methods

### Cell culture

TK6 cells were cultured in RPMI 1640 with GlutaMAX (Gibco), 1.8 mM sodium pyruvate (Gibco), 10% FBS (Gibco) and 100 U/ml penicillin/streptomycin (Gibco). Cells were maintained between 0.05 and 1.0 × 10^6^ cells/ml at 37°C in 5% CO_2_. HEK293ET cells were cultured in IMDM with GlutaMAX supplement (Gibco), 10% FBS (Gibco) and 100 U/ml penicillin/streptomycin (Gibco). Cells were maintained between 5 and 90% confluence at 37°C with 5% CO_2_. Cells were tested regularly for mycoplasma contamination using MycoAlert (Lonza), with thanks to the MRC Laboratory of Molecular Biology Media Kitchen for providing the testing service.

The generation of specific Cas9/CRISPR-assisted gene disruptions and complementation are described in detail in the Supplementary Methods, with reagents listed in Supplementary Tables S1–S5.

### Genome-wide CRISPR/Cas9 knockout screen

3.24 × 10^8^ cells were transduced with titrated lentivirus bearing the Human Improved Genome-wide Knockout CRISPR library (57), with a target MOI of 0.3. Transduction efficiency was assessed 3 days post-transduction by flow cytometry. Cells were passaged every day, splitting to 0.4 × 10^6^ cells/ml maintaining a minimum of 1000× coverage per sgRNA. Transduced cells were selected using 0.5 μg/ml puromycin (Sigma-Aldrich) between days 3 and 10 post-transduction. At day 7 post-transduction, each culture was divided into two parallel cultures. One of these comprised the cisplatin-challenged screen, where cells were passaged with 0.25 μM cisplatin between days 7–14, adding fresh medium with 0.25 μM cisplatin when cells are passaged.

Genomic DNA was extracted from 2 × 10^8^ cells using the Gentra Puregene kit (Qiagen). 96 PCR reactions were set up per condition using Q5 Hot Start High-Fidelity 2X Master Mix (NEB) with 5 μg genomic DNA per reaction using the primers gRNA-HiSeq-SE50 F1/R1 (18 cycles, *T*_A_: 61°C). Eight sets of 12 reactions per condition were concentrated using QIAquick PCR Purification Kits (Qiagen) and analysed on a 2% SYBR Safe (1X)-stained (Invitrogen) agarose gel. The product was excised, purified using a QIAquick Gel Extraction Kit (Qiagen) and quantified using a NanoDrop 2000 (Thermo Scientific). Per condition, 8 PCR reactions were set up using KAPA HiFi HS RM (Roche) using 10 ng of the first PCR reaction product as a template per reaction, the P5 primer and one of P7 indexing primers (6 cycles, *T*_A_ 60°C). Sets of 4 PCR products were pooled and concentrated using a QIAquick PCR Purification Kit (Qiagen). Products were electrophoresed on a Novex 8% TBE polyacrylamide gel (Invitrogen), stained using 1× SYBR Safe (Invitrogen) and excised. Gel fragments were shredded by centrifuging at 15 000 g for 5 minutes in gel-breaker tubes created by piercing the base of 0.5 ml tubes with a 26G needle, collecting shredded fragments in 1.5 ml tubes. 400 μl 0.3 M NaCl was added to the fragments and rotated end-over-end for two hours at room temperature. Gel fragments were removed by centrifugation at 10 000 g for 2 min in a Costar Spin-X Centrifuge Tube Filter (Corning), collecting the flow-through. 20 μg glycogen was added to the flow-through and ethanol precipitation performed. The pellet was dissolved in 20 μl 10 mM Tris–HCl, pH 8.0. Libraries were quantified using a Qubit dsDNA HS Assay Kit (Invitrogen), pooled, and sequenced using either an Illumina HiSeq 4000 with 10% PhiX spike-in or an Illumina NextSeq 2000 with no PhiX spike-in. In both cases, single-end 50 bp reads were obtained, using the custom sequencing primer U6-Illumina-seq2. Primers were ordered as PAGE-purified oligonucleotides from Sigma-Aldrich. Raw sequencing data are deposited in Zenodo (doi: 10.5281/zenodo.8232516). Primers utilised are listed in Supplementary Table S5.

Alignment of the sgRNA variable sequences was performed using the MAGeCK count command (version 0.5.8) (Li *et al.*, 2014). The MaGeCK test command was used to compare sgRNA abundance between sequenced libraries. Volcano plots were produced using p-values and log_2_-fold-change values calculated using MAGeCK test. Aligned sgRNA read-counts are provided in Supplementary Table S6 and the output of the analysis for the untreated and cisplatin-treated screens provided in Supplementary Tables S7 and S8.

### Colony survival assays

5 ml viscous culture medium consisting of 11.9 g/l RPMI 1640 powder with l-glutamine supplement (Gibco), 2 g/l sodium hydrogen carbonate (Sigma-Aldrich), 10 g/l 4000 cP methylcellulose (Thermo Fisher) and 100 U/l penicillin/streptomycin (Gibco) was added to wells of 6-well plates. Plates were equilibrated at 37°C, 5% CO_2_ for a minimum of 1 hour. Cells were treated with the indicated doses of DNA damaging agents and then 20 000, 2000 or 200 cells added to a well for each cell line/condition. Plates were incubated at 37°C, 5% CO_2_ until well-defined colonies were visible. Percentage survival relative to untreated cells was calculated to assess clonogenic survival.

### Growth curves

Cells were plated at 0.05 × 10^6^ viable cells/ml in wells of a 6-well plate. At 24-h intervals, 600 μl culture was taken and viable (trypan blue-negative) cells were counted using a ViCell XR (Beckman Coulter). Where stated, doubling time was calculated from the reciprocal of the gradient of the linear portion of a graph of log_2_([viable cells]) against time.

### DNA fibre spreading

1 million cells for each condition were seeded in 1 ml TK6 medium in wells of a 6-well plate. At the start of the labelling period, 50 μM IdU was added to each well. 20 min after addition of IdU, 100 μM CldU was added to each well. Alongside addition of CldU, either 100 μM cisplatin was added, an equal volume of 0.9% NaCl was added (untreated control) or cells were irradiated with 25 J m^−2^ UV-C. 60 min following addition of IdU, 10 ml ice-cold PBS was added to arrest replication. Cells were washed once in ice cold PBS and resuspended in a final volume of 1 ml ice cold PBS, centrifuging at 500 g, 4°C. 3 μl cells were added to a microscope slide and allowed to dry for 5 min. 7 μl spreading buffer (0.5% SDS in 200 mM Tris–HCl, pH 5.5, with 50 mM EDTA) was added to form a single drop. After 3 min, the slide was tilted at 20° allowing the drop to run down the slide. Following this, the slides were fixed for 10 min using 3:1 methanol:glacial acetic acid and stored at –20°C until staining.

DNA on the fixed slides was denatured using 2.5 M HCl for 45 min. Slides were then dehydrated through sequential 1 minute washes with 70%, 90% and 100% ethanol and allowed to dry. Slides were blocked using BlockAid (Invitrogen) under coverslips in a humidified box. Slides were then stained with the following antibodies: 1:5 mouse anti-BrdU (BD Bioscience, 347580) and 1:25 rat anti-BrdU (Abcam, ab6326) for 45 min; 1:25 rabbit anti-mouse AF594 (Invitrogen, A-11062) and 1:25 chicken anti-rat AF488 (Invitrogen, A-21470) for 30 min; 1:25 donkey anti-rabbit AF594 (Invitrogen, A-21207) and 1:25 goat anti-chicken AF488 (Invitrogen, A-32931) for 30 min; 1:25 anti-ssDNA (DHSB, AB_10805144) for 45 min; goat anti-mouse AF647 (Invitrogen, A-21236) for 30 min and finally chicken anti-goat AF647 (Invitrogen, A-21469) for 30 min. Antibodies were diluted as indicated in BlockAid and added to the slides under coverslips, incubating for the stated period at room temperature in a humidified chamber. Between antibody incubations, slides were washed 3 × 3 min with PBS. Following the final set of washes, slides were dehydrated through sequential 1 min washes with 70%, 90% and 100% ethanol and allowed to dry. Slides were then mounted in 10% PBS, 90% glycerol under coverslips, sealed with nail polish and stored at –20°C until imaging.

Slides were imaged using a Nikon Eclipse TE2000-E with a Hamamatsu ORCA-Flash4.0 LT^+^ cooled CMOS camera, using the 60× objective lens. The length of IdU and CldU labelled tracts were measured at single replication forks, scoring only replication tracts on unbroken fibres. The ratio of CldU/IdU tract length was calculated for each fork and used to monitor replication fork progression.

### Sister chromatid exchange assays

TK6 cells were incubated for 28 h with 20 μM BrdU. 8 hours before harvesting, 10 μM cisplatin was added to treated samples. To arrest cells in metaphase, 0.5 μM nocodazole was added 3 h before harvesting to all samples. Cells were harvested by centrifugation at 250 g for 5 min and resuspended in 75 mM KCl. After 20 min incubation at room temperature, cells were centrifuged at 250 g for 5 min at 4°C. Cells were then sequentially centrifuged (250 g, 5 min, 4°C) and then gently resuspended in progressively smaller volumes of ice-cold 3:1 methanol:glacial acetic acid using wide-bore pipette tips (5 ml, 3 ml, 1 ml and finally 0.5 ml). Fixed cells were stored at 4°C until use.

Metaphase spreads were produced by gently resuspending the samples and then dropping 25 μl droplets onto prechilled, humified slides from a height of 0.5 m using wide-bore pipette tips. After incubation at 65°C for 1 h, slides were washed in 2× SSC for 5 min and then stained using 2 μg/ml Hoechst 33258 diluted in 2× SSC for 15 min. Crosslinking was then performed for 30 min using a CL-1000 Ultraviolet Crosslinker (UVP). Slides were dehydrated by washing twice for 5 min each in 70% ethanol followed by a single 5-min wash using 96% ethanol. Slides were then dried at 65°C for 5 min, washed once for 5 min in PBS and then the DNA was denatured by incubating slides in 0.07 M NaOH for 2 min. Slides were washed 2 × 5 min with PBS and then blocked for 1 h using PBS with 1% BSA and 0.5% Tween 20. The slides were then stained overnight using Purified Mouse Anti-BrdU (BD Biosciences, 347580) diluted 1:1 in PBS with 3% BSA and 0.5% Tween 20. Slides were washed 3 × 5 min using PBS with 1% BSA and 0.5% Tween 20. Slides were then stained with rabbit anti-Mouse IgG AF594 (Invitrogen, A-11062) diluted 1:500 in PBS with 1% BSA and 0.5% Tween 20. Following a 6-hour incubation, slides were washed 3 × 15 min using PBS with 1% BSA and 0.5% Tween 20. Slides were then stained for 15 min at room temperature using 5 μg/ml Hoechst 33342. Slides were washed 3 × 10 min with PBS and once for 5 min with H_2_O. Slides were then mounted in 10% PBS, 90% glycerol under coverslips, sealed with nail polish and stored at –20°C until imaging. Imaging was performed using a Biotek Lionheart FX microscope, using the 60× objective. The number of observable SCE events and the total number of scorable chromosomes per metaphase spread were both counted and used to calculate a normalised value for the SCE per metaphase spread. All incubation steps were performed at room temperature unless otherwise stated.

## Results

### The genetic interactions of PRIMPOL from genome-wide CRISPR/Cas9 knockout screens

To determine the pathways that promote cell survival in the absence of PRIMPOL, we used CRISPR/Cas9 to introduce frameshift deletions within both alleles of exon 5 of the *PRIMPOL* gene to generate *primpol* (i.e. *PRIMPOL^−/−^*) cells in the human TK6 cell background (Supplementary Figure S1A). The selected clone did not express PRIMPOL protein (Supplementary Figure S1B). TK6 cells are frequently used to study DNA replication and the response to DNA damage in human due to their karyotypic stability (58,59) and their expression of functional p53 (60). To facilitate CRISPR/Cas9 screens, expression cassettes allowing stable expression of Cas9, YFP and blasticidin S deaminase (BSD) were inserted into the *AAVS1* safe harbour locus (61) of both TK6 WT and *primpol* cells (Supplementary Figure S1C and D). Cas9 expression was confirmed by monitoring YFP expression (Supplementary Figure S1E) and demonstrating efficient loss of MHC class I from the cell surface following introduction of sgRNAs targeting the *B2M* gene by transduction (Supplementary Figure S1F and G).

**Figure 1.**
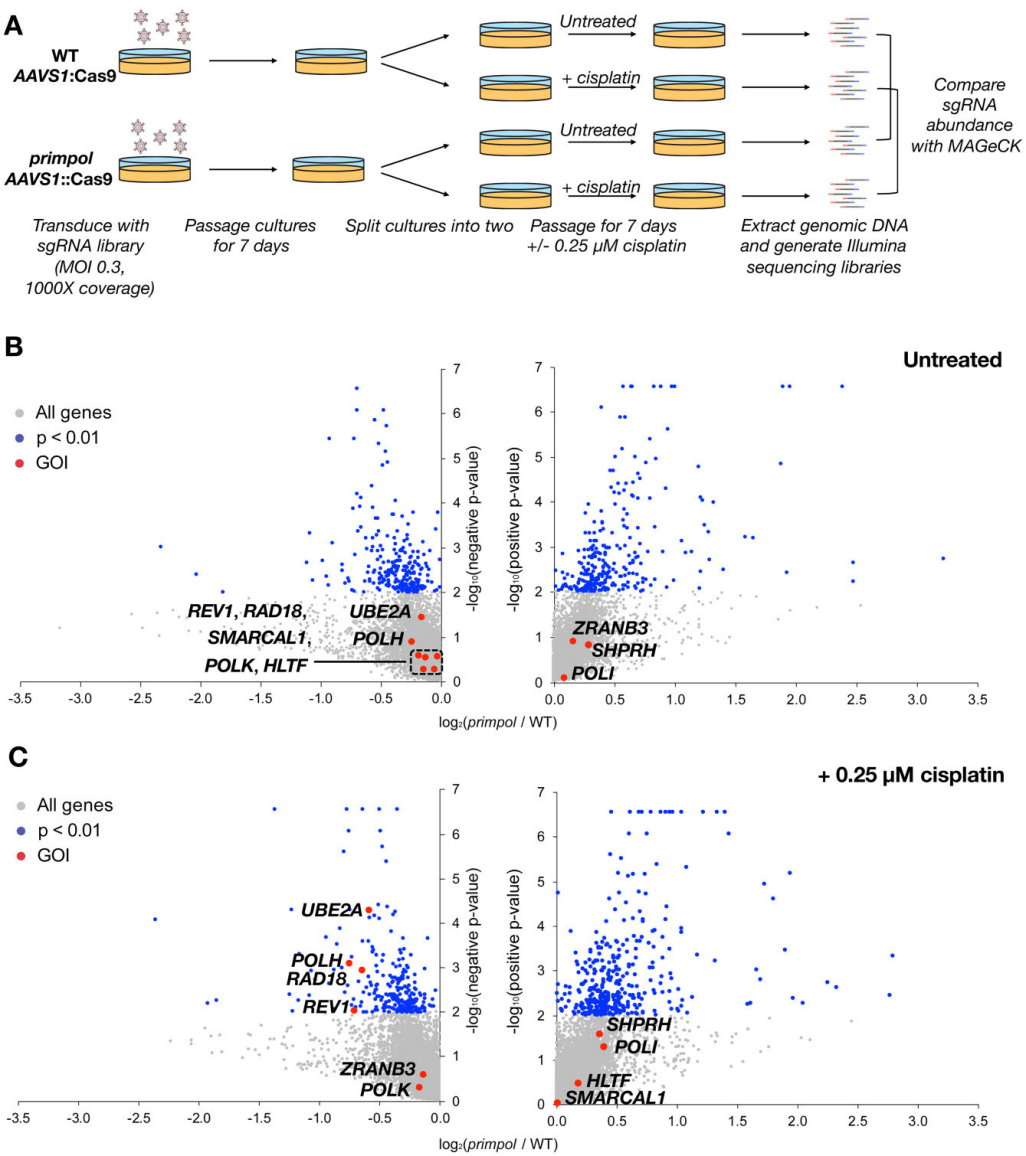
CRISPR/Cas9 knockout screens to assess the genetic interactions of DNA damage tolerance pathways with *PRIMPOL*. (**A**) Summary of the screen design. (**B**) Volcano plots showing genes with sgRNAs depleted or enriched in *primpol* versus WT cells in untreated conditions. (**C**) Volcano plots showing genes with sgRNAs depleted or enriched in *primpol* versus WT cells following 7 days continuous treatment with 0.25 μM cisplatin. In (B, C), blue points represent genes whose sgRNA depletion reaches *P* < 0.01. Key genes of interest for this study are highlighted in red. See Supplementary Tables S6–S8 for a full list of results. *n* = 2 biological replicates. GOI = gene of interest.

To perform the genome-wide CRISPR/Cas9 KO screens, we transduced these TK6 *AAVS1*::Cas9 and TK6 *primpol AAVS1*::Cas9 cells with lentivirus bearing the Human Improved Genome-wide Knockout CRISPR library (57). Following bulk transduction by spinoculation, the WT and *primpol* pools were passaged for 7 days, the cultures split into two and passaged for a further 7 days (Figure 1A). For both cell lines, one culture was left untreated, while the second was maintained in 0.25 μM cisplatin. Cisplatin was chosen as model DNA damaging agent due to its induction of both intra- and inter-strand DNA crosslinks (62,63), its engagement of multiple DNA damage tolerance pathways (64) and its documented role in triggering an adaptive response to DNA damage underpinned by PRIMPOL upregulation (47).

Fourteen days post-transduction, genomic DNA was extracted, and Illumina sequencing libraries generated. For the two biological replicates performed, which demonstrated good correlation (*R*^2^> 0.85) (Supplementary Figure S2), the computational tool MAGeCK (65) was used to compare sgRNA representation between the WT and *primpol* libraries for both untreated and cisplatin-treated conditions. The complete results are presented in Supplementary Tables S6–S8.

In this work, we focus on the interaction of PRIMPOL with the DNA damage tolerance pathways and it is these interactions that we explore in detail in this study. Before doing this, it is worth commenting on some of the top ‘hits’ that confer cisplatin resistance or sensitivity to cells in the absence of PRIMPOL (Supplementary Figure S3A and B). The three top genes that provide resistance to cisplatin in the absence of PRIMPOL are CDKN1A (p21), CDK2 and TP53, which enforce checkpoint activation following DNA damage (66). At the other end of the screen, guides to several relatively poorly characterised genes appeared as significantly enriched in *primpol* mutants versus WT cells. These included EDC4 (67) and BOD1L (68), which have been implicated in replication stress and recombination and the PAXIP1 (PTIP)/PAGR1 complex, which is linked to the DNA damage response (69,70). However, when we made new knockouts of these genes in both wild type and *primpol* mutant TK6 cells, we could detect no change in doubling time (Supplementary Figure S3C–G), which would have been predicted by the results of the screen. We elected not to further pursue these potential interactions in detail.

Following cisplatin challenge, sgRNAs targeting the DNA damage tolerance genes *REV1*, *POLH*, *RAD18* and *UBE2A* were all depleted in *primpol* cells compared with WT with *P*< 0.01 (Figure 1B and C). Surprisingly, guides targeting the fork reversal factors (*SMARCAL1*, *HLTF*, *ZRANB3, SHPRH*) or components of the Fanconi Anaemia pathway were neither enriched nor depleted (Tables S6–S8), despite previous studies suggesting a genetic interaction between some of these factors and PRIMPOL in transformed cell lines (47,50). To explore these results further we generated new mutants in otherwise completely wild type TK6 cells.

### Loss of PCNA K164 ubiquitination creates a broad requirement for PRIMPOL in suppressing sensitivity to DNA damaging agents

**Figure 2.**
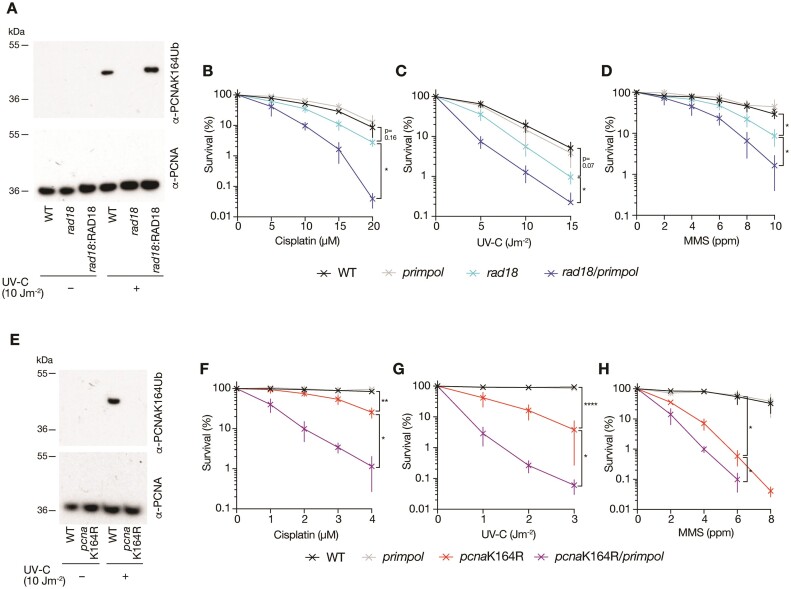
Loss of PCNA K164 ubiquitination reveals a broad requirement for PRIMPOL in suppressing sensitivity to DNA damaging agents. (**A**) PCNA K164 ubiquitination monitored with an anti-PCNAK164Ub antibody 4 h following irradiation with 10 Jm^−2^ UV-C in *rad18* knockout TK6 and *rad18* complemented with ectopic expression of human RAD18. (B–D) Colony survival of WT (black), *primpol* (grey), *rad18* (cyan) and *rad18*/*primpol* (blue) TK6 cells following treatment with cisplatin (**B**), UV-C irradiation (**C**) and MMS (**D**). (**E**) PCNA K164 ubiquitination in *pcnaK164R* TK6 cells monitored with an anti-PCNAK164Ub antibody 4 h following irradiation with 10 J m^−2^ UV-C. (F–H) Colony survival of WT (black), *primpol* (grey), *pcna*K164R (red) and *pcna*K164R/*primpol* (purple) TK6 following treatment with cisplatin (**F**), UV-C irradiation (**G**) and MMS (**H**). For clonogenic survival assays, cisplatin and MMS treatments were performed using the indicated doses for 1 hour, UV-C irradiation was performed as a single pulse. Percentage clonogenic survival was calculated relative to equal numbers of untreated cells plated in parallel. Results are from *n* = 3 to 6 biological replicates, with mean and standard deviation plotted. p-values are calculated using Brown-Forsythe and Welch ANOVA tests followed by two-tailed unpaired *t* tests with Welch's correction: *P< 0.05; **P< 0.005.

PCNA ubiquitination plays an important role downstream of PRIMPOL promoting filling the post-replicative gaps created by PRIMPOL-dependent repriming (52,53). However, the appearance of *RAD18* and *UBE2A* as ‘hits’ in our screen under cisplatin challenge suggested that, additionally, PCNA K164 ubiquitination plays an important role in promoting cell survival when PRIMPOL is lost. To confirm this observation, we used CRISPR/Cas9 assisted homologous recombination to generate new TK6 mutants lacking PRIMPOL (Supplementary Figure S4) and the ability to ubiquitinate PCNA K164, *rad18* and *pcna*K164R (Supplementary Figure S5). We confirmed loss of PCNA K164 monoubiquitination stimulated by UV-C irradiation in both the *rad18* and *pcna*K164R cells by Western blot (Figure 2A and E), although, in some experiments (e.g. Figure 7A), residual PCNA ubiquitination was observed in the absence of RAD18, confirming previous observations in DT40 cells (71). Additionally, we confirmed that the increased sensitivity of the *rad18* mutant to UV-C irradiation was reversed by expression of human RAD18 (Supplementary Figure S5E–G) and generated *rad18/primpol* and *pcna*K164R/*primpol* double mutants (Supplementary Figure S5H–K).

As expected, *rad18* and *pcna*K164R cells exhibited hypersensitivity to a range of DNA damaging agents, including UV-C radiation, the alkylating agent methylmethane sulphonate (MMS), the quinoline 4-nitroquinoline-1-oxide (4NQO) and interstrand crosslinking agent mitomycin C (MMC) (Figures 2 and S6). In line with the results of the CRISPR/Cas9 screens, removal of PRIMPOL in the *rad18* and *pcna*K164R backgrounds further increased the sensitivity of cells to both acute (Figures 2 and S6) and chronic cisplatin treatment (Supplementary Figure S7). The sensitivity of *pcna*K164R *primpol* cells to cisplatin could be rescued to the level seen in the *pcna*K164R mutant by expression of WT but not catalytically inactive or primase-incompetent PRIMPOL (Supplementary Figure S8) confirming that this increased sensitivity was due to loss of the repriming function of PRIMPOL. As previously observed in DT40 cells (71) loss of RAD18 does not lead to the same degree of sensitivity as mutation of the target ubiquitination site in PCNA at K164 (Figures 2 and S5). This is consistent with the existence of secondary E3 ligases capable of PCNA ubiquitination (71–73) and roles of other PCNA K164 modifications, such as polyubiquitination and SUMOylation.

### Loss of PRIMPOL and PCNA K164, but not RAD18, suppresses damage-induced sister chromatid exchange

**Figure 3.**
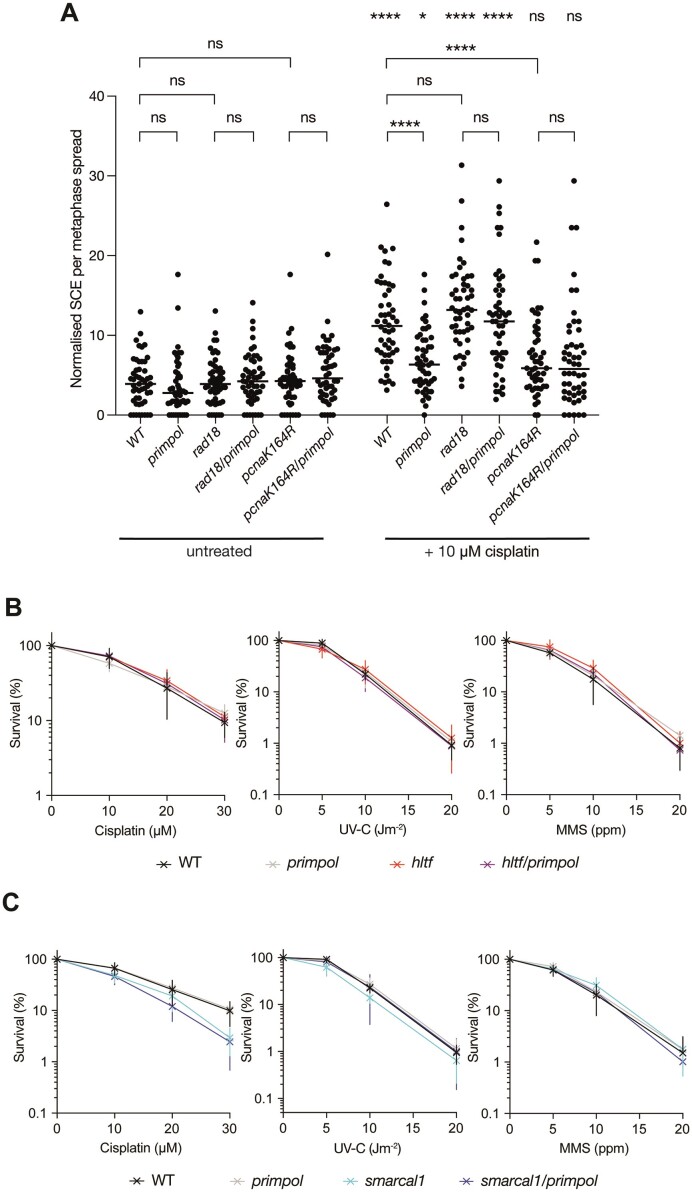
Damage-induced sister chromatid exchange requires PRIMPOL and PCNA K164 modification. (**A**) Sister chromatid exchange in a panel of TK6 cell lines lacking the capacity for PCNA ubiquitination and/or PRIMPOL was assessed following treatment with 10 μM cisplatin for 8 h. Horizontal bar indicates the median value. *P*-values calculated with one-way ANOVA test: *P< 0.05; **P< 0.005, ***P< 0.0005, ****P< 0.00005. For each condition, 50 metaphases are counted with the number of SCEs corrected for the number of scorable chromosomes. (**B**) Colony survival of WT (black), *primpol* (grey), *hltf* (red) and *hltf*/*primpol* (purple) TK6 cells following treatment with cisplatin (left panel), UV-C irradiation (central panel) and MMS (right panel). (**C**) Colony survival of WT (black), *primpol* (grey), *smarcal1* (cyan) and *smarcal1/primpol* (blue) TK6 cells following treatment with cisplatin (left panel), UV-C irradiation (central panel) and MMS (right panel). Cisplatin and MMS treatments were performed using the indicated doses for 1 h, UV-C irradiation was performed as a single pulse. Statistical testing utilised Brown-Forsythe and Welch ANOVA tests—no statistically-significant difference (*P* ≥ 0.05) in clonogenic survival for all cell lines for the highest dose of DNA damaging agent tested. *n* = 3 to 4 biological replicates with mean and standard deviation plotted.

PRIMPOL-mediated repriming leads to gaps which can be filled by homologous recombination promoting sister chromatid exchange (SCE). Thus, disruption of PRIMPOL reduces damage-induced SCE (51) suggesting that PRIMPOL-repriming directs gaps to a recombination-based pathway of gap filling. Consistent with these observations, we observed a reduction in cisplatin-induced SCE in *primpol* TK6 cells. Further, these damage-induced SCE were dependent on PCNA ubiquitination as the suppression of SCE observed following loss of PRIMPOL was epistatic with the PCNA K164R mutation (Figure 3A). This epistasis for SCEs suggests that loss of the PCNA ubiquitination-dependent recombination/template switch is unlikely to explain the increased sensitivity of the *pcna*K164R/*primpol* double mutant. Further, following disruption of SMARCAL1 or HLTF (Supplementary Figure S9), which have been implicated in PCNA ubiquitination-dependent damage tolerance by template switching, we observed no increased sensitivity irrespective of PRIMPOL status (Figures 3 and S10). This observation was surprising given the probable lethality of disruption of both PRIMPOL and SMARCAL1 in chicken DT40 cells (47), a point we discuss further below. Interestingly, we also observed that, in contrast to the *pcna*K164R mutant, *rad18* cells retained higher levels of damage-induced SCEs irrespective of PRIMPOL status (Figure 3A). We observed a similar distinction between the effects of deleting RAD18 and its target lysine in PCNA on SCE in DT40 cells (71). This may reflect channelling of lesions or gaps into a classical recombination pathway when RAD18 is absent (71,74).

### PRIMPOL-deficient cells rely on specific TLS polymerases in a damage-specific manner

**Figure 4.**
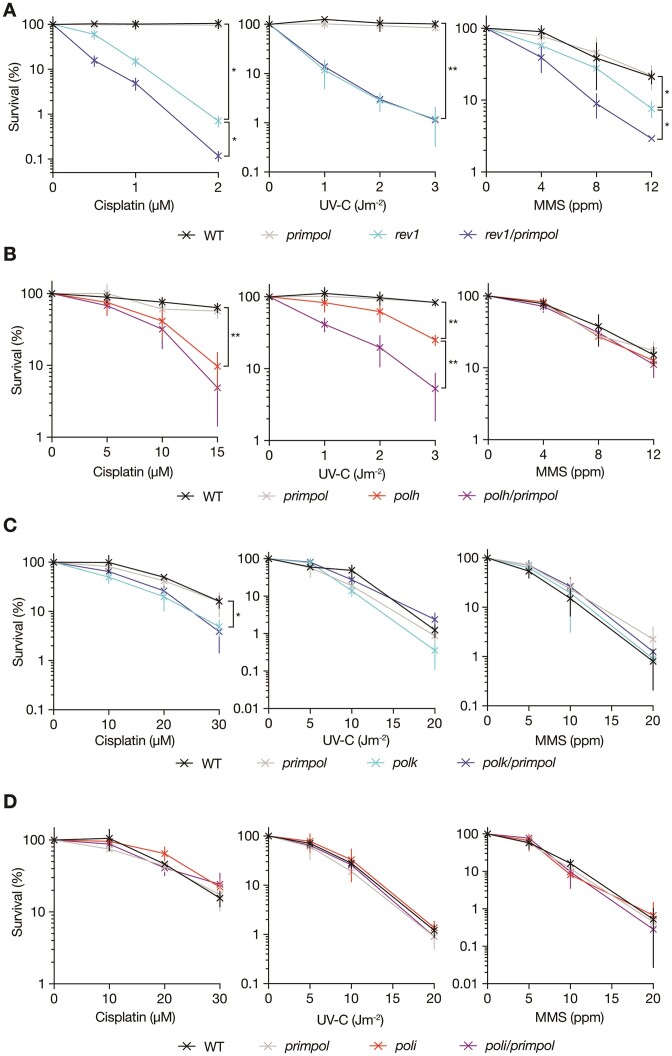
PRIMPOL suppression of sensitivity to DNA damage in Y-family polymerase mutants is agent- and polymerase-specific. Clonogenic survival assays assessing the sensitivity of TK6 cells lacking REV1 (**A**), POLη (**B**), POLκ (**C**) or POLτ (**D**) +/– PRIMPOL to cisplatin (left panels), UV-C irradiation (central panels) and MMS (right panels). Cisplatin and MMS treatments were performed using the indicated doses for 1 h, UV-C irradiation was performed as a single pulse. Results are from *n* = 3 to 4 biological replicates, with mean and standard deviation plotted. *P*-values are calculated using Brown-Forsythe and Welch ANOVA tests followed by two-tailed unpaired *t* tests with Welch's correction: *P < 0.05; **P < 0.005, ***P < 0.0005, ****P < 0.00005. If not shown, there is no statistically significant difference (p ≥ 0.05) in clonogenic survival at the highest dose of DNA damaging agent between the WT and TLS polymerase single mutant, or between the TLS single and the double mutant cell lines.

We therefore turned to the role of TLS in supporting PRIMPOL-deficient cells, motivated by the identification of both REV1 and POLη in the initial CRISPR/Cas9 dropout screen under cisplatin challenge (Figure 1C). We generated *rev1, polh, polk* and *poli* mutant cells and double mutants with *primpol* and confirmed the disruptions by PCR and Western blotting (Supplementary Figure S11 and S12). In contrast to the broad interaction of PRIMPOL deficiency with defective PCNA ubiquitination, cells lacking the TLS polymerases POLη and REV1 were further sensitised by loss of PRIMPOL, but only to specific DNA damaging agents (Figures 4 and S13). Thus, loss of PRIMPOL alongside REV1 further sensitised cells only to cisplatin and MMS (Figures 4A and S13A), while loss of PRIMPOL alongside POLη further sensitised cells to UV-C (Figure 4B & S13B). However, TK6 cells lacking either *POLK* (Figures 4C and S13C) or *POLI* (Figures 4D and S13D) exhibited no significant hypersensitivity to any of these agents, as previously reported (75), and this was not modulated by loss of PRIMPOL. The observed hypersensitivity could be reversed by complementation by ectopic expression of tagged versions of the proteins in question (Figures S14 and S15). The contribution of REV1 was dependent on its C-terminus, but not catalytic activity (Supplementary Figure S15F) as previously observed in DT40 cells (21) and that of PRIMPOL on both its catalytic activity and zinc finger, which is necessary for its repriming activity (Supplementary Figure S15C and I).

**Figure 5.**
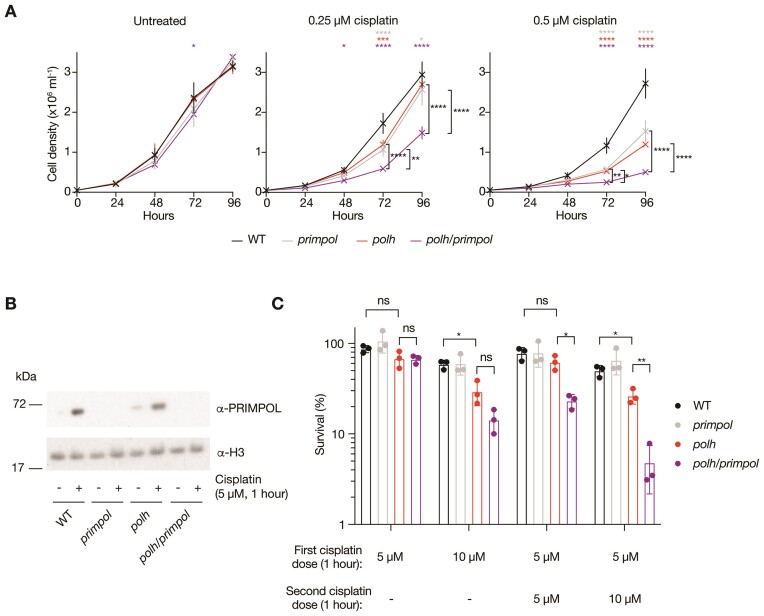
Chronic cisplatin treatment or repeated acute cisplatin exposures increases the requirement for PRIMPOL in POLη-deficient cells. (**A**) TK6 *polh/primpol* cells show reduced proliferation upon chronic exposure to 0.25 or 0.5 μM cisplatin, as assessed using growth curves. Results are from *n* = 3 biological replicates, with mean and standard deviation plotted. p-values are calculated using two-way ANOVA with Tukey correction: *P< 0.05; **P< 0.005, ***P< 0.0005, ****P< 0.00005. (**B**) Western blots showing upregulation of PRIMPOL protein in both TK6 WT and *polh* cells, 24 h following 1 h exposure to 5 μM cisplatin. Representative of *n* = 3 biological replicates. (**C**) TK6 *polh/primpol* cells show reduced colony forming efficiency when exposed to repeated acute treatments with cisplatin. Cells were exposed to single or repeated (24 h later) acute 1-h exposures to cisplatin. Percentage clonogenic survival is calculated relative to equal numbers of untreated cells plated in parallel or, when two cisplatin exposures are performed, cells only exposed to the initial cisplatin dose. Results are from *n* = 3 biological replicates, with mean and standard deviation plotted. *P*-values are calculated using Brown-Forsythe and Welch ANOVA tests followed by two-tailed unpaired *t* tests with Welch's correction: *P< 0.05; **P< 0.005.

Interestingly, despite *POLH* appearing as a hit in the CRISPR/Cas9 dropout screen, somewhat surprisingly PRIMPOL loss did not significantly increase the sensitivity of *polh* cells to acute cisplatin treatment (Figure 4B). However, the screen was performed in the presence of continuous cisplatin treatment. When we assessed the sensitivity of *polh*, *primpol* and *polh/primpol* cells to chronic cisplatin treatment using growth curves, more closely mimicking the chronic treatment regimen used in the CRISPR/Cas9 screen, combined loss of PRIMPOL and POLη clearly increased cisplatin sensitivity (Figure 5A). The increased requirement for PRIMPOL when chronic versus acute treatment regimens are used is consistent with the previously described adaptive response to cisplatin mediated by upregulation of PRIMPOL mRNA and protein (47). In agreement with this idea, exposure to a single acute dose of cisplatin leads to upregulation of PRIMPOL protein (Figure 5B). Further, two acute doses of cisplatin applied 24 h apart, reveals that loss of PRIMPOL further sensitises *polh* cells to cisplatin in clonogenic survival assays (Figure 5C).

### Maintenance of fork progression in the face of DNA damage depends on TLS but is PCNA K164R ubiquitination independent

**Figure 6.**
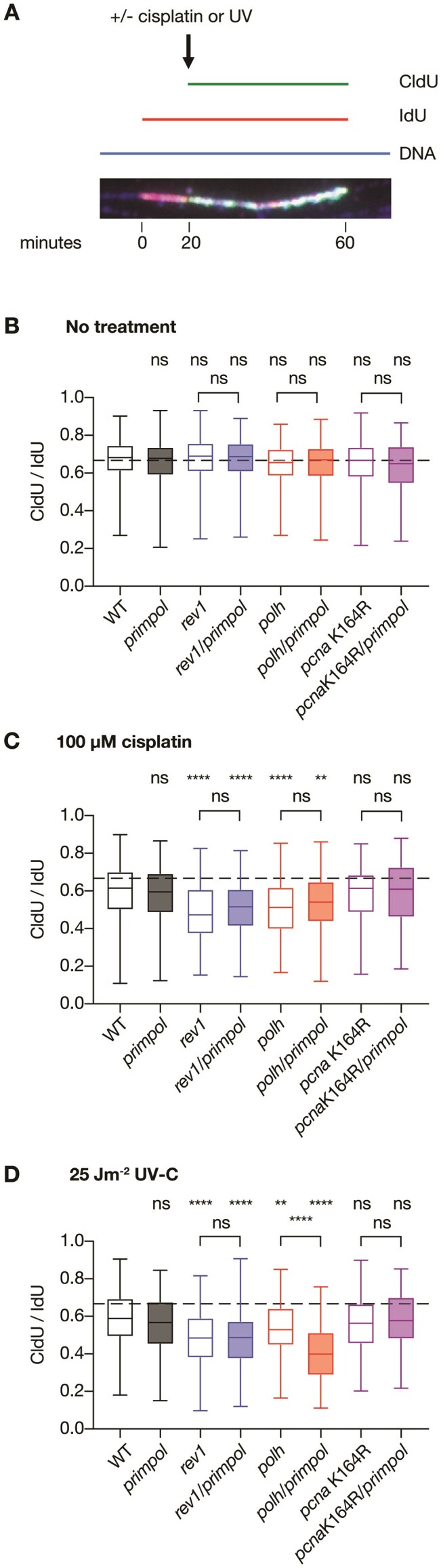
Assessment of fork progression rates when PRIMPOL is absent alongside REV1, POLη or in a *pcna*K164R background. DNA replication tracks were labelled in a panel of TK6 cell lines through addition of 50 μM IdU for 20 min, followed by addition of 100 μM CldU for a further 40 min (**A**). At 20 min, when CldU was added, cells were either left untreated (**B**), 100 μM cisplatin added (**C**) or irradiated with 25 Jm^−2^ UV-C irradiation (**D**). Fork progression was assessed from the ratio of CldU/IdU tract length for intact replication forks on unbroken DNA fibres, as assessed by staining using an anti-DNA antibody. Results are pooled across *n* = 3 to 4 biological replicates, giving a minimum of 200 scored replication forks per cell line per condition. *P*-values were calculated using the Kruskal–Wallis test with Dunn's correction: *P< 0.05; **P< 0.005, ***P< 0.0005, ****P< 0.00005. A representative image of DNA fibre staining is provided in Figure S16.

**Figure 7.**
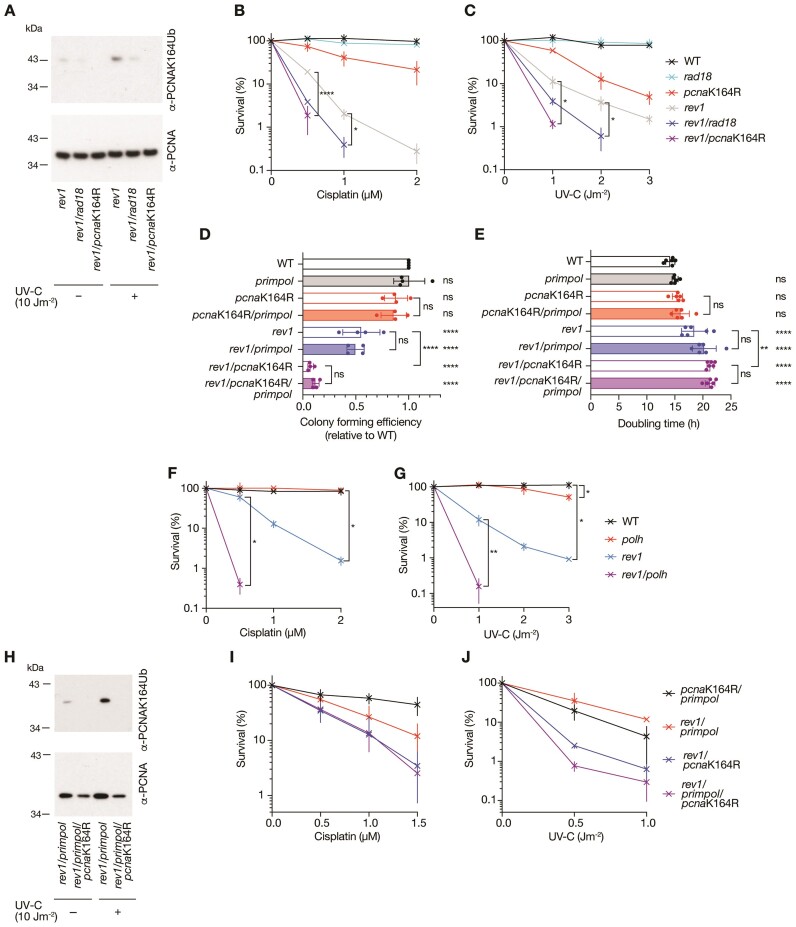
The genetic relationship of PRIMPOL to REV1 and PCNAK164. (**A**) PCNA K164 ubiquitination monitored with an anti-PCNAK164Ub antibody 4 h following irradiation with 10 Jm^−2^ UV-C in *rev1*/*rad18* and *rev1/pcna*K164R mutant TK6 cells. (B, C) Colony survival of WT (black), *rad18* (cyan), *pcna*K164R (red), *rev1* (grey), *rev1/rad18* (blue) and *rev1/pcna*K164R (purple) TK6 cells following treatment with cisplatin (**B**) and UV-C irradiation (**C**). (**D**) Colony forming efficiency of *rev1* and *pcna*K164R mutants in combination with loss of PRIMPOL. Colony formation is normalised to the colony forming efficiency of WT cells. Results are from *n* = 4 biological replicates, with mean and standard deviation plotted. *P*-values are calculated using ordinary one-way ANOVA tests with the Holm–Šídák correction: *P< 0.05; **P< 0.005, ***P< 0.0005, ****P< 0.00005. (**E**) Doubling time of *rev1* and *pcna*K164R mutants in combination with loss of PRIMPOL. Results are from *n* = 6 biological replicates, with mean and standard deviation plotted. *P*-values are calculated using ordinary one-way ANOVA tests with the Holm–Šídák correction: *P< 0.05; **P< 0.005, ***P< 0.0005, ****P< 0.00005. (F, G) Colony survival of WT (black), *polh* (red), *rev1* (cyan), *rev1/polh* (purple) TK6 cells following treatment with cisplatin (**F**) and UV-C irradiation (**G**). (**H**) PCNA K164 ubiquitination monitored with an anti-PCNAK164Ub antibody 4 hours following irradiation with 10 Jm^−2^ UV-C in *rev1/primpol* and *rev1/primpol/pcna*K164R mutant TK6 cells. (I, J) Colony survival of *pcna*K164R/*primpol* (black), *rev1*/*primpol* (red), *rev1/pcna*K164R (blue) and *rev1/primpol/pcna*K164R (purple) TK6 cells following treatment with cisplatin (**I**) and UV-C irradiation (**J**). For clonogenic survival assays to assess the sensitivity to cisplatin, treatment was performed using the indicated doses for 1 h, UV-C irradiation was performed as a single pulse. Results are from *n* = 3 to 5 biological replicates, with mean and standard deviation plotted. *P*-values are calculated using Brown-Forsythe and Welch ANOVA tests followed by two-tailed unpaired *t* tests with Welch's correction: *P< 0.05; **P< 0.005, ***P< 0.0005, ****P< 0.00005.

Our previous work in DT40 cells has suggested that lesion bypass by TLS can be temporally and genetically separated into that taking place at the fork, which we termed on-the-fly TLS and that depends on the non-catalytic function of REV1, and post-replicative TLS, which is coordinated by PCNA ubiquitination (15). Therefore, we next assessed the contribution of REV1, PCNA K164 and PRIMPOL to maintenance of DNA synthesis rates in the face of template DNA damage using DNA fibre spreading assays (Figure 6). As we observed in DT40 cells (15), loss of the TLS polymerases REV1 and POLη led to impaired overall DNA synthesis rates following UV-C or cisplatin treatment compared with wild type cells. In contrast, the *pcna*K164R mutant exhibited behaviour indistinguishable from wild type. Emphasising these distinct contributions of REV1 and PCNA K164 ubiquitination to DNA damage tolerance, and mirroring previously obtained results in DT40 cells (15), *rev1/rad18* and *rev1/pcna*K164R TK6 cells (Supplementary Figure S17) are considerably more sensitive to these agents than the respective single mutants (Figure 7A–C).

We did not observe any impact of loss of PRIMPOL on unperturbed replication fork progression in TK6 cells (Figure 6B). Notably, however, PRIMPOL loss further impaired fork progression in UV-C treated (Figure 6D), but not cisplatin-treated *polh* cells (Figure 6C) consistent with the proposal that POLη is deployed early at UV impediments and recently published results demonstrating the importance of PRIMPOL in maintaining fork progression in the absence of POLη-mediated on-the-fly TLS (41,54,55). In contrast, we did not observe a further decrease in fork rates when PRIMPOL is deleted in cells lacking REV1, suggesting that direct bypass of UV lesions by POLη may differ from other lesions that may require REV1. Interestingly, a *rev1/polh* double mutant (Supplementary Figure S18) is very significantly more sensitive to UV light and cisplatin than either single mutant (Figure 7F, G**)**. This would be surprising if all POLη action is coordinated by REV1 but may also be explained by the close interaction and shepherding of POLη to ubiquitinated PCNA by RAD18 (10), which may also be REV1-independent.

Taken together our data suggests that loss of PRIMPOL is backed up by both on-the-fly TLS and post-replicative gap filling and lesion bypass and that it acts at the nexus of the two pathways. To test this further, we created *rev1/pcna*K164R/*primpol* triple mutants (Supplementary Figure S17). *rev1/pcna*K164R cells are already extremely sensitive to DNA damage (Figure 7B and C), clone poorly (Figure 7D) and grow slowly (Figure 7E). However, the additional disruption of PRIMPOL was not inviable and added not at all or minimally to the already severe phenotypes (Figure 7D, E, H–J), suggesting that use of PRIMPOL is unlikely to engage further mechanisms not mediated through PCNA K164 or REV1, such as a PCNA ubiquitination-independent classical recombination pathway.

## Discussion

### A model for the role of PRIMPOL-dependent repriming in damage tolerance

The principal role of DNA damage tolerance is to ensure that replication stalling lesions are safely returned to double stranded DNA for repair while simultaneously limiting the formation of single stranded DNA caused by uncoupling of DNA unwinding and DNA synthesis. Previous work has suggested that the mechanisms deployed to achieve this vary as a function of time from when the lesion is initially encountered. Thus, the C-terminus of REV1 plays an important role in coordinating translesion synthesis at the fork and PCNA ubiquitination plays a key role in coordinating post-replicative gap filling (15). Consistent with these two mechanisms backing each other up, DT40 *rev1* and *pcna*K164R mutants are not epistatic for survival following DNA damage (15), a relationship recently been reported in mice (17) and recapitulated in the present study.

Here, we provide genetic insights into how PRIMPOL fits within the handover between these two mechanisms of bypass. Loss of PRIMPOL significantly impacts the ability of cells to survive DNA damage when they lack the ability to ubiquitinate PCNA and, in a lesion specific manner, when REV1 or POLη are not available at the fork, showing that PRIMPOL interacts with both layers of damage tolerance. Further, *rev1/pcna*K164R/*primpol* triple mutant cells are viable, despite the significant synthetic sickness of the combined loss of REV1 and PCNA K164 (Figure 7) suggesting that PRIMPOL is not required to engage a further significant pathway beyond those controlled by REV1 or PCNA K164 modification, for instance classical HR.

**Figure 8.**
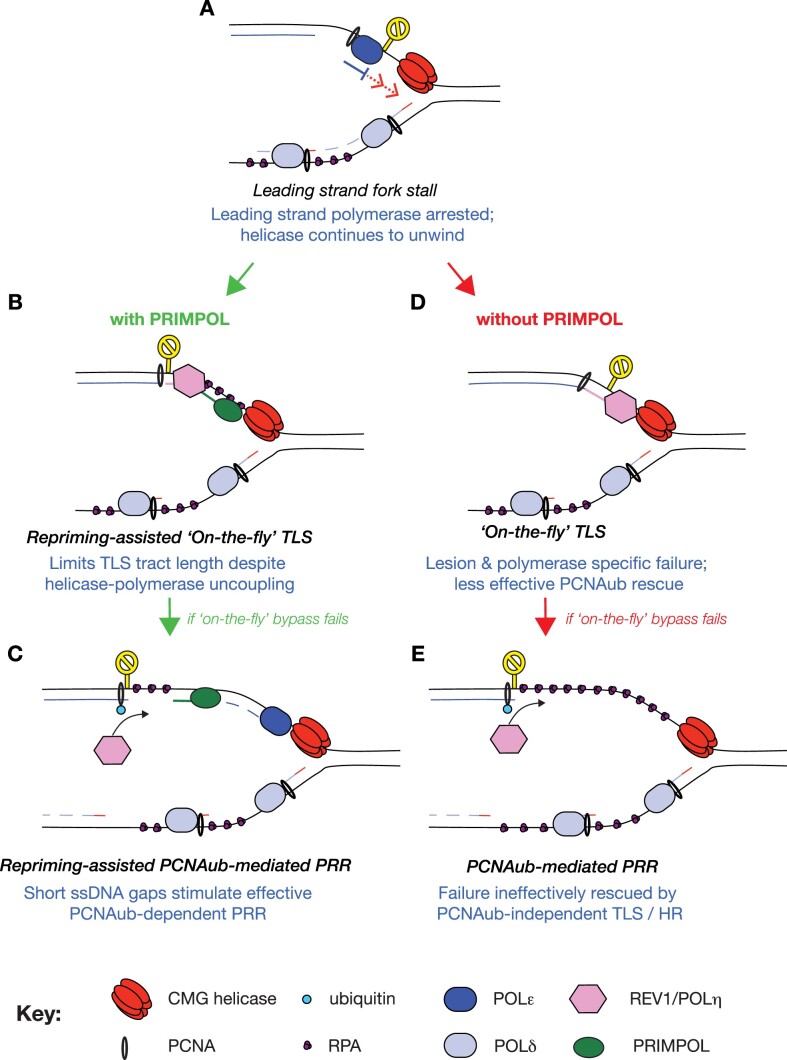
PRIMPOL-mediated repriming facilitates the interaction between on-the-fly TLS and PCNAub-dependent post-replicative damage bypass. (**A**) Following arrest of leading strand synthesis, the CMG helicase can continue to unwind at a slower rate than during processive replication, creating a region of single stranded DNA, the length of which will depend on time. (B, C) RPA binding to this single stranded DNA will facilitate recruitment of PRIMPOL, which restricts the length of gap needing to be filled by REV1/Polh-dependent on-the-fly TLS (**B**) or PCNA ubiquitination-dependent post-replicative gap filling (**C**). (**D**,**E**) In the absence of PRIMPOL, on-the-fly and post-replicative mechanisms would have longer gaps to deal with limiting their effectiveness and ability to compensate for each other.

It is likely that the phenotypes resulting from PRIMPOL-dependent repriming originate primarily from the leading strand given the constitutive Okazaki priming that takes place on the lagging strand. In considering a model for these interactions of PRIMPOL-dependent repriming (Figure 8) it is instructive to consider the sequence of events that follow stalling of a leading strand polymerase as being determined by a ‘timer’ specified by the replicative CMG helicase (Figure 8A). The CMG helicase is thought to continue to unwind DNA following polymerase arrest, albeit at a slower rate than normal (43). As the helicase continues to unwind DNA, in the absence of synthesis it will gradually increase the length of ssDNA in between the stalled polymerase and itself. If the lesion can be bypassed immediately, as exemplified by the direct bypass of some lesions by POLδ (76,77), the rapid recruitment of POLη to CPDs (55) or REV1-dependent TLS (15), this should prevent any significant helicase polymerase uncoupling and, if rapid enough, obviate the need for RPA recruitment. Loss of these on-the-fly mechanisms will lead to lesion specific or general defects in fork progression monitored by DNA fibre assays.

However, it is unlikely that such TLS mechanisms are uniformly rapid and thus further uncoupling and ssDNA exposure may take place while they are being deployed. Once sufficient ssDNA is exposed for RPA to be loaded, a number of molecular events can take place. ssDNA/RPA forms a platform for recruitment of checkpoint proteins (78) but is also needed for both recruitment of PRIMPOL (79) and RAD18 (11), which mediates PCNA ubiquitination. It remains unclear how competition between these mechanisms at a stalled fork is regulated. Importantly for the model we are proposing, however, loss of PCNA ubiquitination does not have a significant impact on progression of DNA synthesis in DNA fibre assays, suggesting that it is deployed late. PRIMPOL deletion alone in TK6 cells does not affect fibre tract length, monitored with DNA counterstaining, compared to wild type cells either in the presence or absence of DNA damage, in contrast to previous reports in DT40 (41) and HeLa cells (39). Indeed, even combined loss of pcnaK164 ubiquitination and PRIMPOL does not slow replication tract elongation following DNA damage (Figure 6). However, deletion of PRIMPOL clearly reduced DNA fibre tract length in *polh* mutants following UV damage (Figure 6 and 55, 54, 41). This suggests that PRIMPOL is normally sufficiently rapidly recruited in *polh* cells to have a visible impact on ameliorating the slowing of tract elongation, in contrast to PCNA ubiquitination. Thus, we can tentatively suggest that recruitment of PRIMPOL is likely to generally precede PCNA ubiquitination.

Early deployment of PRIMPOL will have the effect of restricting the length of the gap formed if on-the-fly bypass is delayed (Figure 8B). In turn this will have a number of benefits. It will restrict the number of nucleotides needing to be incorporated during on-the-fly TLS. Further, should on-the-fly TLS fail, PRIMPOL will limit the effects of extensive fork uncoupling, and chromosomal instability (80) and epigenetic instability (48,81) will be avoided. However, the gaps formed by PRIMPOL-dependent repriming are still flagged by PCNA ubiquitination (Figure 8C), which, in turn can promote their resolution through post-replicative TLS (52,53) or post-replicative recombination, which can lead to sister chromatid exchange (Figure 3A and 51). When PCNA K164 modification is not an option, cells likely rely heavily on ongoing attempts at PCNA K164-independent on-the-fly TLS, which, we propose is less likely to succeed and/or be toxic when gap length is not restricted by repriming (Figure 8D and E).

### The contribution of fork reversal factors to DNA damage tolerance in non-transformed cells

Deleting the fork reversal factors HLTF and SMARCAL1 did not itself substantially increase cell sensitivity to DNA damaging agents, nor did it reveal a requirement for PRIMPOL in suppressing sensitivity to DNA damaging agents, in contrast to loss of the ability to modify PCNA, which has been linked to fork reversal/template switching in both yeast and higher eukaryotes (reviewed in (5,6). This is somewhat surprising considering the previous observations that loss of PRIMPOL promotes fork reversal, whilst deletion of fork reversal factors increases PRIMPOL-mediated repriming (36,47,52). Why does loss of both pathways not sensitise TK6 cells to DNA damaging agents? One possibility is that there is sufficient redundancy between fork reversal mechanisms in these genetically close to normal cells and that it would be necessary to disrupt multiple fork reversal factors to observe an effect. Another possibility is that immortalised but non-transformed cells, like TK6 rely on fork reversal very little. This may explain why, in contrast to our failed attempts to generate *smarcal1/primpol* DT40 mutants (47), neither gene was identified in the screen reported here and that it was straightforward to generate this mutant in TK6. Nonetheless, the PRIMPOL adaptive response to chronic damage is still present in TK6 (Figure 5). Increased PRIMPOL levels induced by exposure to cisplatin improves the ability of the cells to tolerate subsequent exposure to the agent, especially in the absence of POLη, showing that even without significant reliance on SMARCAL1, upregulation of PRIMPOL improves tolerance to damage.

## Supplementary Material

gkad1054_Supplemental_Files

## Data Availability

Sequencing data for the CRISPR screen can be accessed at Zenodo (doi: 10.5281/zenodo.8232516).

## Notes

Present address: Christopher Mellor, Division of Nutritional Sciences, Cornell University, Ithaca, NY 14853, USA.

Present address: Saša Šviković, Astra Zeneca, Genome Engineering, Pepparedsleden 1, 43183 Mölndal, Sweden.
