# Supplementary material for: PRIMPOL ensures robust handoff between on-the-fly and post-replicative DNA lesion bypass
Source: Nucleic Acids Res. 2023 Nov 16;52(1):243–58. doi: 10.1093/nar/gkad1054 (PMC10783524; doi:10.1093/nar/gkad1054)
Supplement: gkad1054_Supplemental_Files [file gkad1054_supplemental_files.zip › Mellor et al SI_R1.pdf]

SUPPLEMENTARY INFORMATION FOR:

PRIMPOL ensures robust handoff between on-the-fly and post-replicative DNA lesion bypass.

Christopher Mellor[1], Joëlle Nassar, Saša Šviković[2] & Julian E. Sale[3]

Division of Protein & Nucleic Acid Chemistry, Medical Research Council Laboratory of Molecular Biology, Francis Crick Avenue, Cambridge, CB2 0QH, UK.

[1]Current address: Division of Nutritional Sciences, Cornell University, Ithaca, NY 14853, USA

[2]Current address: Astra Zeneca, Genome Engineering, Pepparedsleden 1, HC3131, 43183 Mölndal, Sweden

[3]To whom correspondence should be addressed: jes@mrc-lmb.cam.ac.uk

Figure S1: Generation of Cas9-expressing TK6 WT and *primpol* cells

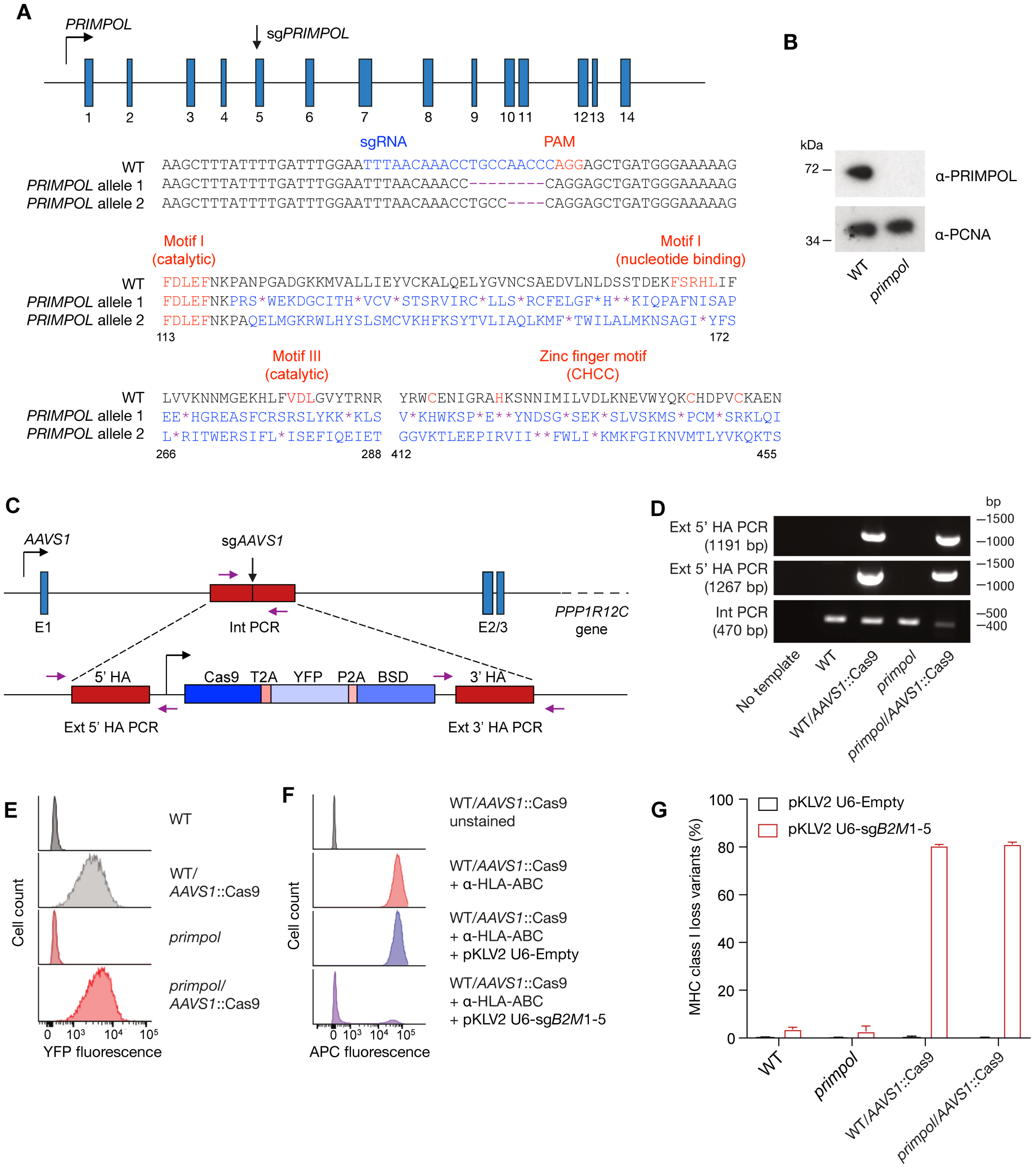

A. CRISPR/Cas9 targeting of exon 5 of the *PRIMPOL* gene introduced 4 nt and 8 nt deletions into the two *PRIMPOL* alleles, as confirmed by Sanger sequencing of a PCR amplicon. **B**. Western blot confirming loss of PRIMPOL protein expression in the TK6 *primpol* cells. **C**. Schematic showing CRISPR/Cas9-assisted insertion of the Cas9-YFP-BSD expression cassette into the safe-harbour *AAVS1* locus. Cas9, YFP and BSD are separated by P2A/T2A self-cleaving peptides. **D**. Confirmation of heterozygous insertion of a Cas9-YFP-BSD expression cassette into TK6 WT and *primpol* cells by genotyping PCR. Amplicons indicated in C. HA = homology arm. **E**. Flow cytometry showing robust YFP expression in

TK6 WT/*AAVS1*::Cas9 and *primpol*/*AAVS1*::Cas9 cells. **F**. Example flow cytometry plots showing loss of cell surface expression of MHC Class I upon transduction of TK6 WT/*AAVS1*:Cas9 cells with sgRNAs targeting *B2M*, as assessed by staining with an APC-labelled anti-HLA-ABC antibody. Representative of three biological replicates. **G**. Quantification of loss of cell surface MHC Class I expression upon transduction of TK6 WT/*AAVS1*::Cas9 and *primpol*/*AAVS1*::Cas9 cells with sgRNAs targeting *B2M* or equivalent empty vector controls. Mean plus standard deviation plotted, n = 3 biological replicates.

Figure S2. Correlation between replicates of the genome-wide CRISPR/Cas9 knockout screens

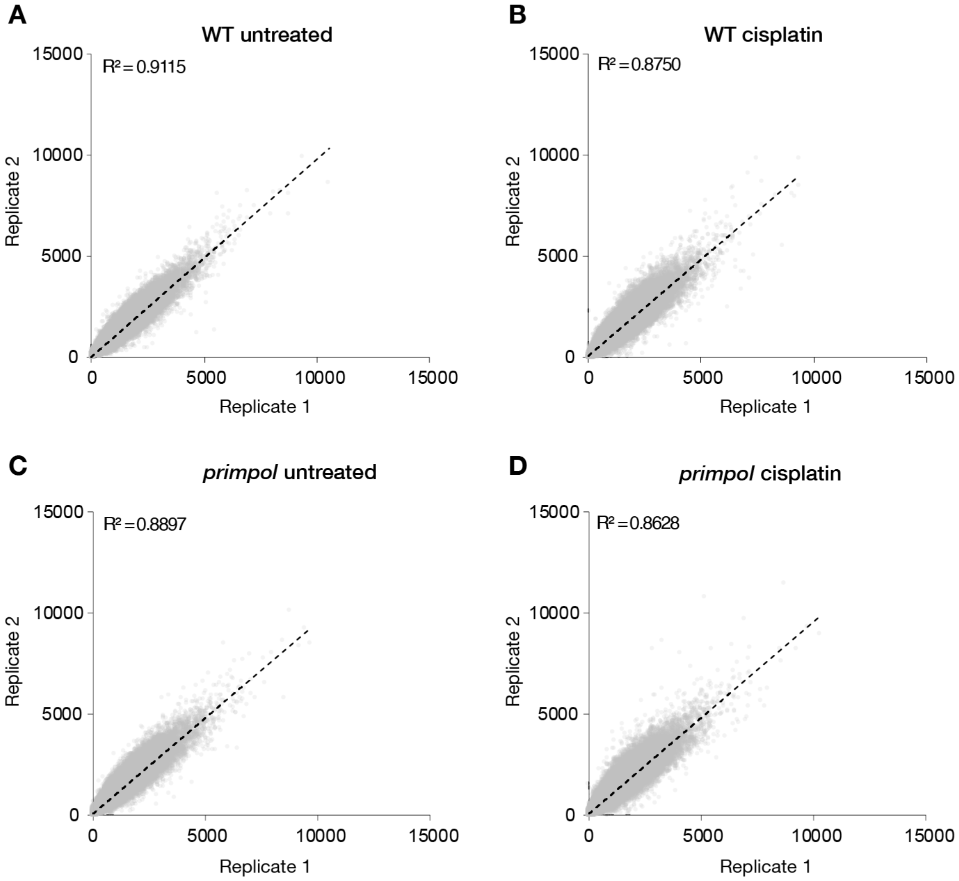

Correlation between normalised sgRNA read counts from the Illumina sequencing libraries generated from the two biological replicates of the genome-wide CRISPR/Cas9 knockout screens. WT untreated (**A**); WT cisplatin-treated (**B**); *PRIMPOL*-/- untreated (**C**) and *PRIMPOL*-/- cisplatin-treated (**D**).

Figure S3. Loss of PRIMPOL does not alter the doubling time of cells lacking BOD1L, EDC4 or PAXIP1.

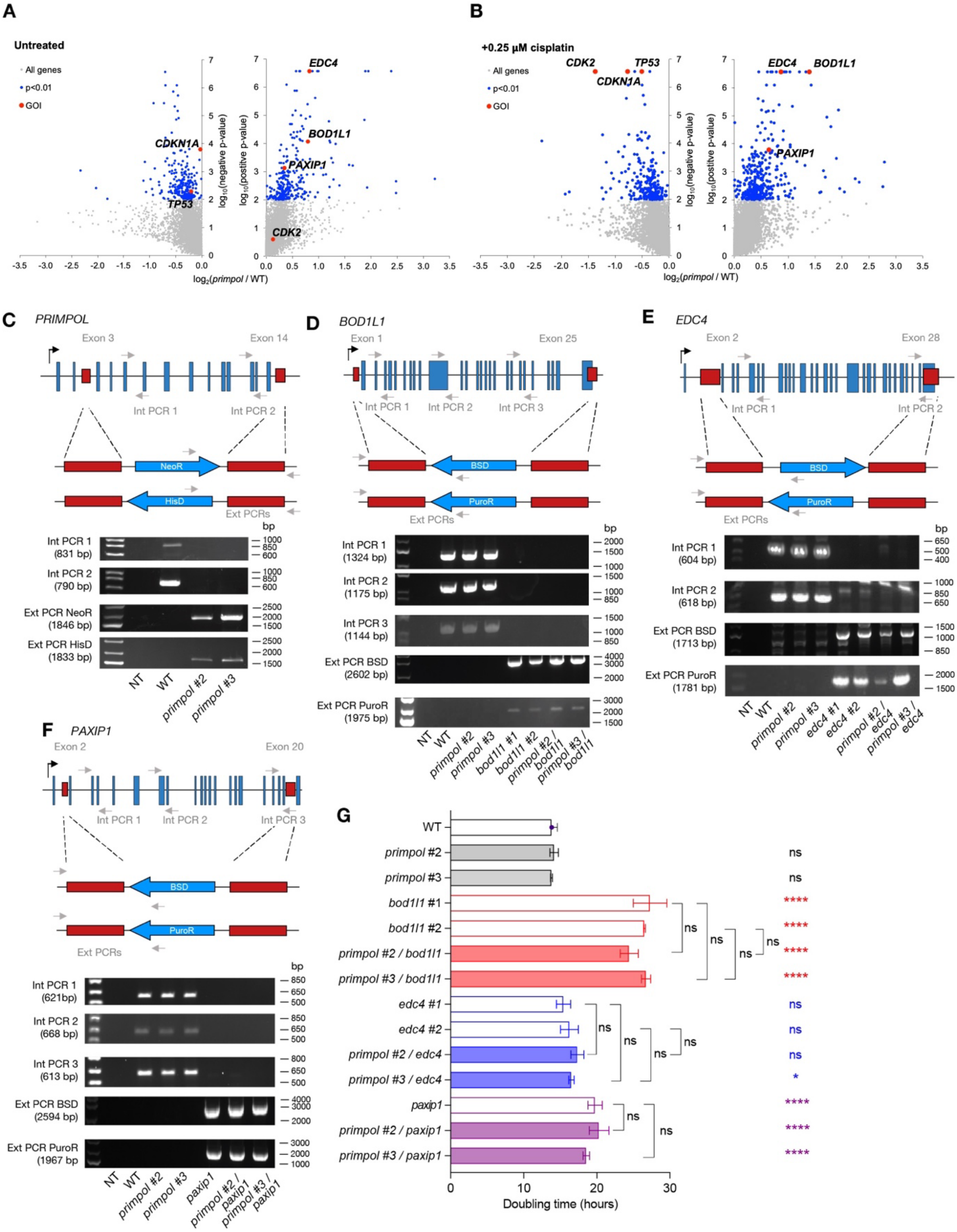

A. Volcano plots showing genes with sgRNAs depleted or enriched in *primpol* versus WT cells in untreated conditions. **B**. Volcano plots showing genes with sgRNAs depleted or enriched in *primpol* versus WT cells following 7 days continuous treatment with 0.25 µM

cisplatin. In A. & B., blue points represent genes whose sgRNA depletion reaches $p < 0.01$. Key genes of interest (GOI) for this study are highlighted in red. See Supplementary Tables S6–S8 for a full list of results. n = 2 biological replicates. **C**. CRISPR/Cas9-assisted introduction of targeting constructs to delete a region of the *PRIMPOL* gene including the entirety of exons 3-14. **D**. CRISPR/Cas9-assisted introduction of targeting constructs to delete a region of the *BOD1L1* gene including the entirety of exons 1-25. **E**. CRISPR/Cas9-assisted introduction of targeting constructs to delete a region of the *EDC4* gene including the entirety of exons 2-28. **F**. CRISPR/Cas9-assisted introduction of targeting constructs to delete a region of the *PAXIP1* gene including the entirety of exons 2-20. Insertion of the targeting constructs into the aforementioned genes was confirmed by genotyping PCR, using PCR amplicons as indicated in the figures. **G**. Doubling time of *bod1l1*, *edc4* and *paxip1* mutants in combination with loss of PRIMPOL. Results are from n=3 biological replicates, with mean and standard deviation plotted. p-values are calculated using ordinary one-way ANOVA tests with the Holm-Šídák correction: *: $p<0.05$; **: $p<0.005$, ***: $p<0.0005$, ****: $p<0.00005$.

Figure S4. An additional TK6 *primpol* mutant for validation of CRISPR/Cas9 screen results.

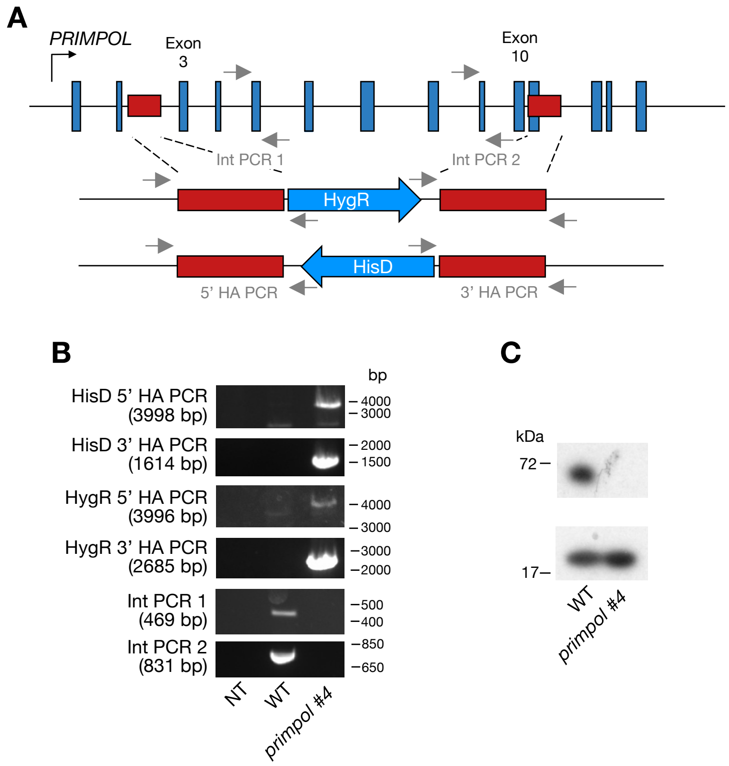

A. CRISPR/Cas9-assisted introduction of targeting constructs to delete a region of the *PRIMPOL* gene including the entirety of exons 3-10. **B**. Insertion of the targeting constructs into the *PRIMPOL* gene was confirmed by genotyping PCR. Amplicons indicated in A. HA = homology arm. **C**. Western blot showing loss of PRIMPOL protein expression in the TK6 *primpol* cells. This TK6 *primpol* clone, #4, is hereafter referred to as TK6 *primpol*.

Figure S5. Generation of TK6 *rad18/primpol* cells and *pcna*K164/*primpol* cells

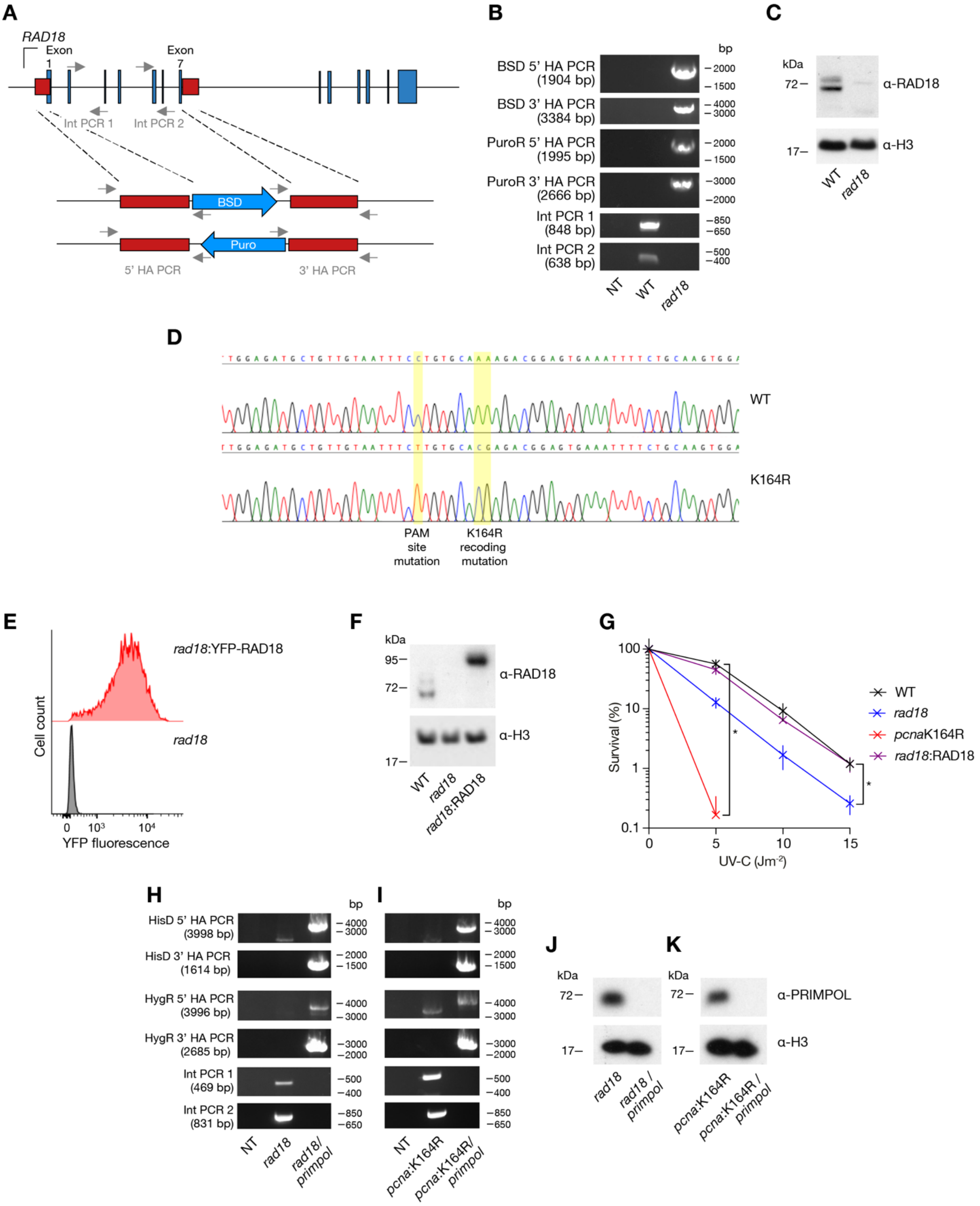

A. CRISPR/Cas9-assisted introduction of targeting constructs to delete a region of the *RAD18* gene including the entirety of exons 2-7. **B**. Insertion of the targeting constructs into the *RAD18* gene confirmed by genotyping PCR. Amplicons indicated in A. HA = homology arm. **C**. Western blot confirming loss of RAD18 protein expression in TK6 *rad18* cells. **D**. Recoding of the *PCNA* gene to introduce the K164R point mutation was achieved by co-

transfecting TK6 cells with Cas9 ribonucleoprotein targeting the PCNA gene alongside a modified ssDNA homology-directed repair donor oligonucleotide. This oligonucleotide encoded three point mutations: two leading to the K164R substitution with a third silent mutation disrupting the PAM site to prevent re-cleavage. Sanger sequencing of a PCR amplicon confirmed the presence of the desired mutations. **E**. Flow cytometry showing robust YFP expression following complementation of TK6 *rad18* cells with YFP-RAD18. **F**. Western blot showing expression of YFP-RAD18 in TK6 *rad18* cells following complementation. **G**. Colony survival assays showing both TK6 *rad18* and *pcna*K164R cells are sensitive to UV-C irradiation. The sensitivity of TK6 *rad18* cells is rescued by complementation. Results are from n = 3 biological replicates, with mean and standard deviation plotted. p-values are calculated using Brown-Forsythe and Welch ANOVA tests followed by 2-tailed unpaired t tests with Welch's correction: *: $p<0.05$. **H & I**. Generation of TK6 *rad18*/*primpol* cells (H) and *pcna*K164/*primpol* cells (I), with targeting of the *PRIMPOL* gene performed as described in Figure S4. Insertion of the targeting constructs into the *PRIMPOL* gene was confirmed by genotyping PCR. **J & K**. Western blots confirming loss of PRIMPOL protein expression in the TK6 *rad18*/*primpol* (J) and *pcna*K164R/*primpol* (K) cells.

Figure S6. ***pcna*K164/*primpol* cells show increased sensitivity to 4NQO and MMC.**

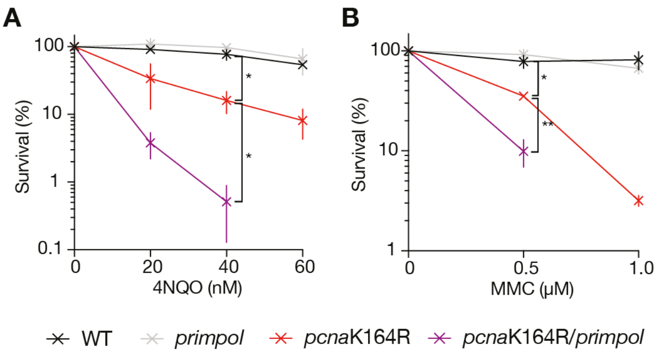

A & B. TK6 *pcna*K164R/*primpol* cells show increased sensitivity to 4NQO (A), and MMC (B) compared to *pcna*K164R cells as assessed by clonogenic survival assays. Treatments were performed for 1 hour. Percentage clonogenic survival was calculated relative to equal numbers of untreated cells plated in parallel. Results are from n = 3 to 4 biological replicates, with mean and standard deviation plotted. p-values are calculated using Brown-Forsythe and Welch ANOVA tests followed by 2-tailed unpaired t tests with Welch's correction: *: $p<0.05$; **: $p<0.005$.

Figure S7. Cells deficient for both PCNA K164 ubiquitination and PRIMPOL show increased sensitivity to chronic cisplatin treatment.

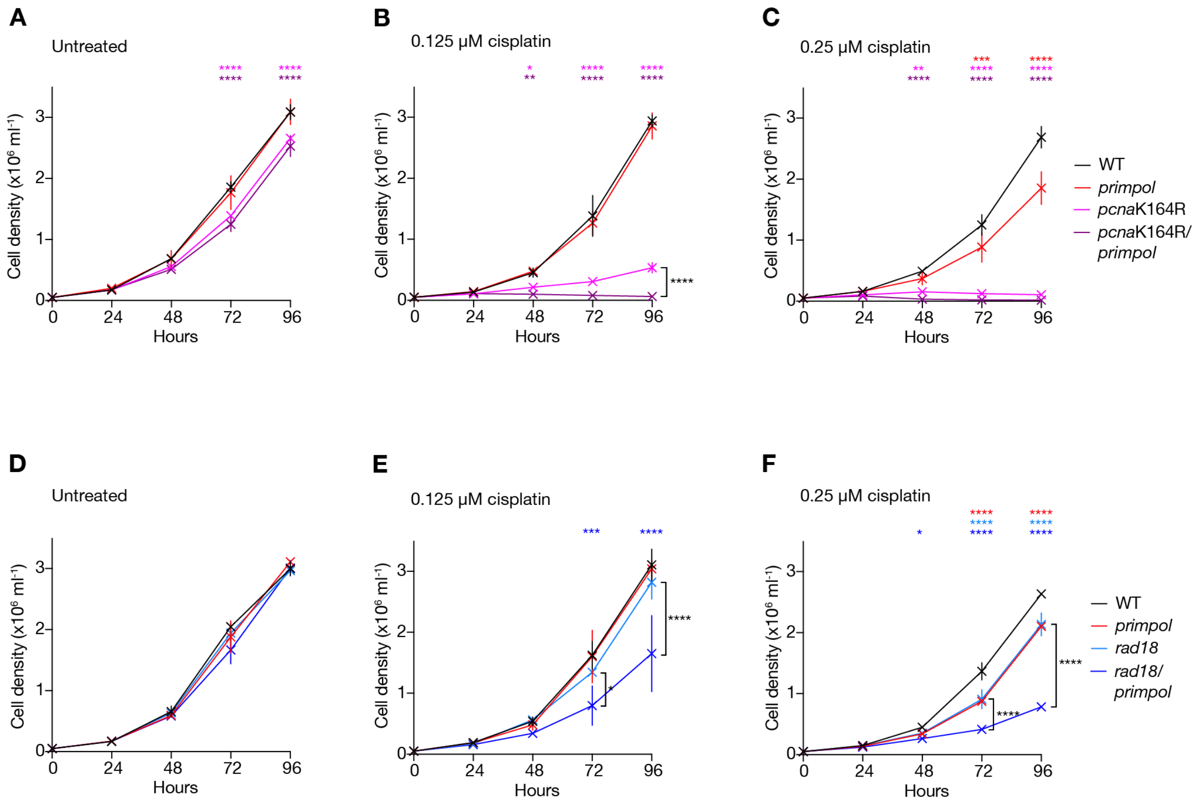

A – C. Growth curves showing relative proliferation of TK6 cells with the PCNA K164R mutation and/or lacking PRIMPOL in untreated conditions (A), or in the presence of continuous exposure to 0.125 µM (B) or 0.25 µM (C) cisplatin. **D – F**. Growth curves showing relative proliferation of TK6 cells lacking RAD18 and/or PRIMPOL in untreated conditions (D), or in the presence of continuous exposure to 0.125 µM (E) or 0.25 µM (F) cisplatin. For all growth curves, results are from n = 3 biological replicates, with mean and standard deviation plotted. p-values are calculated using 2-way ANOVA with Tukey correction: *: $p<0.05$; **: $p<0.005$, ***: $p<0.0005$, ****: $p<0.00005$.

Figure S8. Complementation of *pcna*K164R/*primpol* cells with primase-competent PRIMPOL rescues cisplatin sensitivity to the level of *pcna*K164R cells

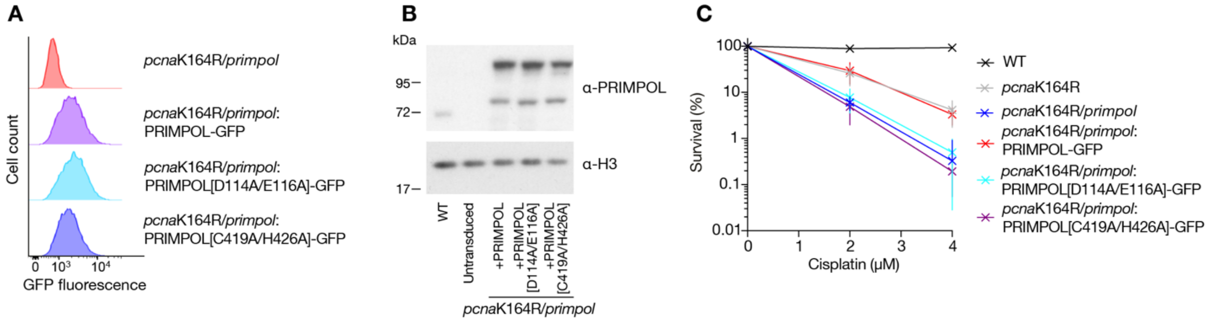

A. Complementation of TK6 *pcna*K164R/*primpol* cells with PRIMPOL variants was performed by transduction with constructs allowing expression of C-terminal GFP-tagged human PRIMPOL variants. PRIMPOL[D114A E116A] is a catalytically-dead variant, whilst the C419A H426A mutation inhibits the primase activity of the enzyme. Flow cytometry showing robust GFP expression in complemented *pcna*K164R/*primpol* cells. **B**. Western blot showing expression of PRIMPOL-GFP variants in complemented cells. **C**. Complementation with WT PRIMPOL but not the D114A E116A or C419A H426A variants rescues the sensitivity of TK6 *pcna*K164R/*primpol* to cisplatin to the level of *pcna*K164R cells, as assessed by clonogenic survival assays. Mean and standard deviation plotted, n = 3 biological replicates.

Figure S9. Generation of TK6 *smarcal1/primpol* cells and *hltf/primpol* cells

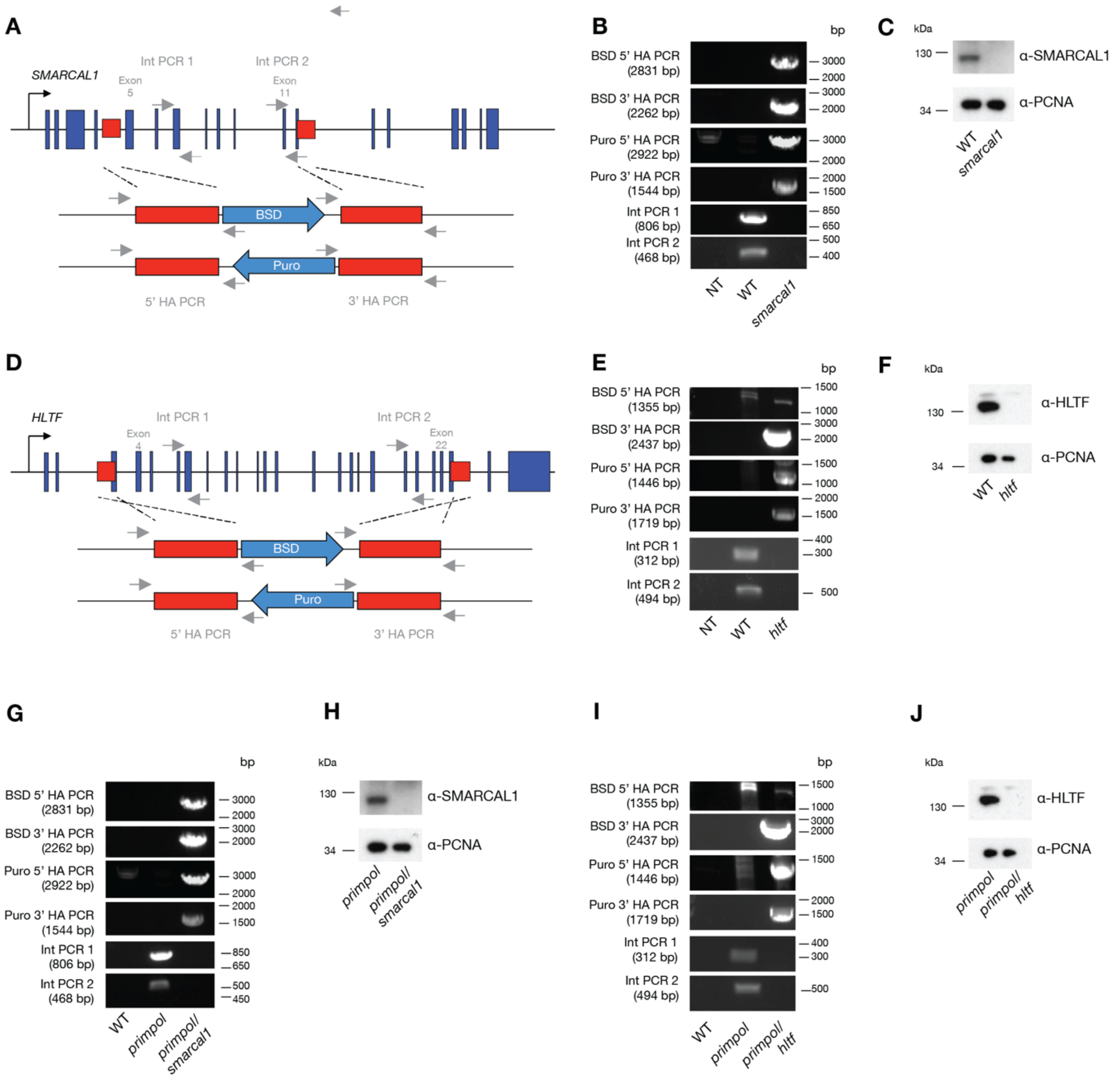

A. CRISPR/Cas9-assisted introduction of targeting constructs to delete a region of the *SMARCAL1* gene including the entirety of exons 5-11. **B**. Insertion of the targeting constructs into the *SMARCAL1* gene following transfection of TK6 WT cells was confirmed by genotyping PCR. **C**. Western blot to confirm loss of SMARCAL1 protein expression in the TK6 *smarcal1* cells. **D**. CRISPR/Cas9-assisted introduction of targeting constructs to delete a region of the *HLTF* gene including the entirety of exons 4-22. **E**. Insertion of the targeting constructs into the *HLTF* gene following transfection of TK6 WT cells was confirmed by genotyping PCR. **F**. Western blot to confirm loss of HLTF protein expression in the TK6 *hltf* cells. **G**. Insertion of the targeting constructs into the *SMARCAL1* gene following transfection of TK6 *primpol* cells was confirmed by genotyping PCR. **H**. Loss of SMARCAL1 protein expression in the TK6 *primpol/smarcal1* cells as confirmed by Western blot. **I**. Insertion of the targeting constructs into the *HLTF* gene following transfection of TK6 *primpol* cells was confirmed by genotyping PCR. **J**. Western blot to confirm loss of HLTF protein expression in

the TK6 *primpol*/*hltf* cells. The genotyping PCR amplicons are indicated on the corresponding map, HA = homology arms.

Figure S10. Combined loss of replication fork factors and PRIMPOL does not sensitise cells to the DNA damaging agents 4NQO and MMC.

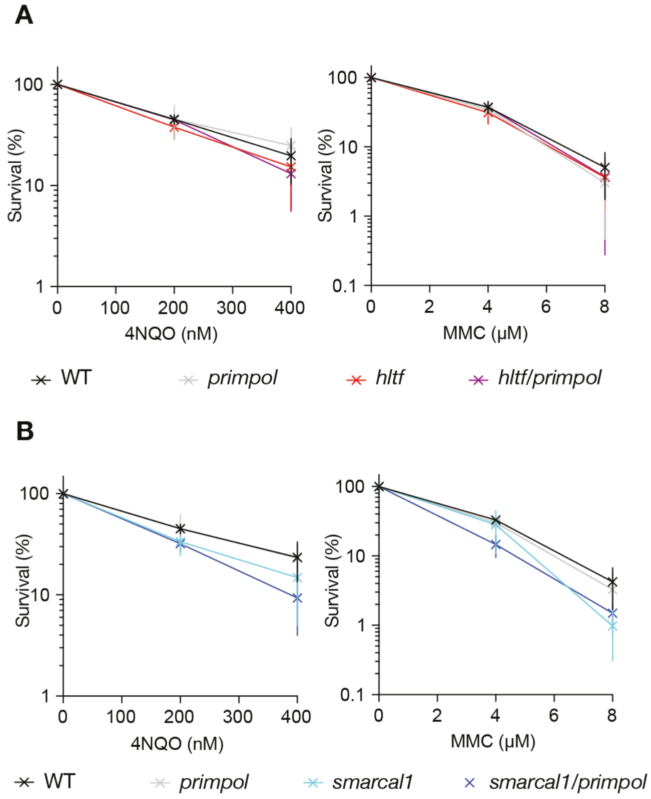

A. TK6 cells lacking HLTF and/or PRIMPOL show no statistically significant difference in sensitivity to 4NQO or MMC compared to WT cells as assessed by clonogenic survival assays. **B**. TK6 lacking SMARCAL1 and/or PRIMPOL cells show no statistically significant difference in sensitivity to 4NQO or MMC compared to WT cells as assessed by clonogenic survival assays. Treatments were performed for 1 hour. Statistical testing utilised Brown-Forsythe and Welch ANOVA tests – no statistically-significant difference ($p \geq 0.05$) in clonogenic survival for all cell lines for the highest dose of DNA damaging agent tested. n = 3 biological replicates with mean and standard deviation plotted.

Figure S11. Generation of Y-family translesion synthesis polymerase knockouts in TK6 cells

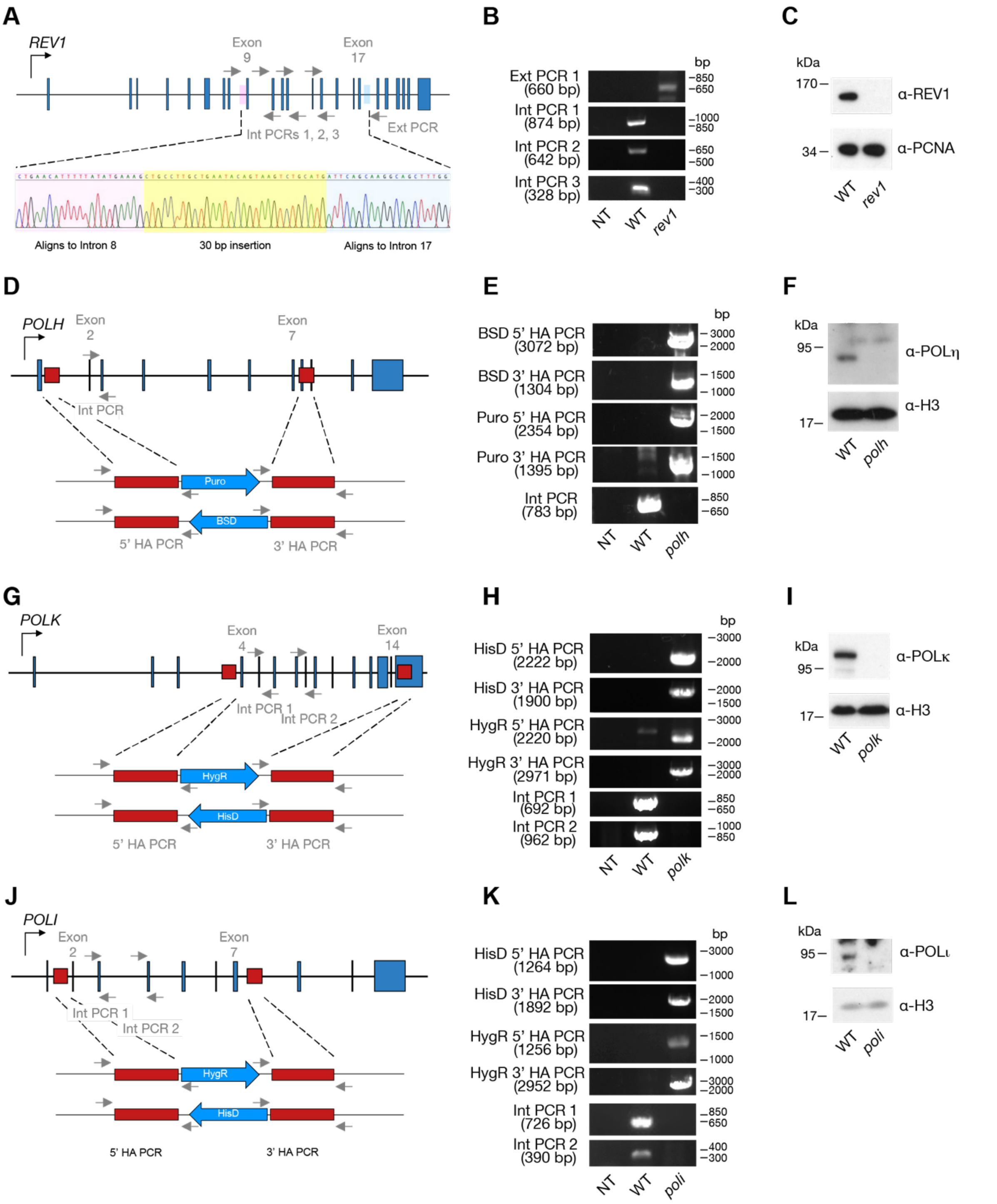

A. TK6 *rev1* cells were generated by CRISPR/Cas9 deletion of a large genomic region including the entirety of exons 9-17. Sanger sequencing of the external PCR (Ext PCR) amplicon indicated in B was used to confirm a biallelic modification, with the sequencing read aligning to regions of introns 8 and 17 either side of a 30-nucleotide insertion. **B**. The deletion within the *REV1* gene was confirmed by genotyping PCR. **C**. Western blot to

confirm loss of REV1 protein expression in the TK6 *rev1* cells. **D**. CRISPR/Cas9-assisted introduction of targeting constructs to delete a region of the *POLH* gene including the entirety of exons 2-7. **E**. Insertion of the targeting constructs into the *POLH* gene was confirmed by genotyping PCR. **F**. Western blot to confirm loss of POLη protein expression in the TK6 *polh* cells. **G**. CRISPR/Cas9-assisted introduction of targeting constructs to delete a region of the *POLK* gene including the entirety of exons 4-14. **H**. Insertion of the targeting constructs into the *POLK* gene was confirmed by genotyping PCR. **I**. Western blot to confirm loss of POLκ protein expression in the TK6 *polk* cells. **J**. CRISPR/Cas9-assisted introduction of targeting constructs to delete a region of the *POLI* gene including the entirety of exons 2-7. **K**. Insertion of the targeting constructs into the *POLI* gene was confirmed by genotyping PCR. **L**. Western blot to confirm loss of POLι protein expression in the TK6 *poli* cells. HA = homology arm.

Figure S12. Generation of TK6 cell lines lacking PRIMPOL and the Y-family polymerases

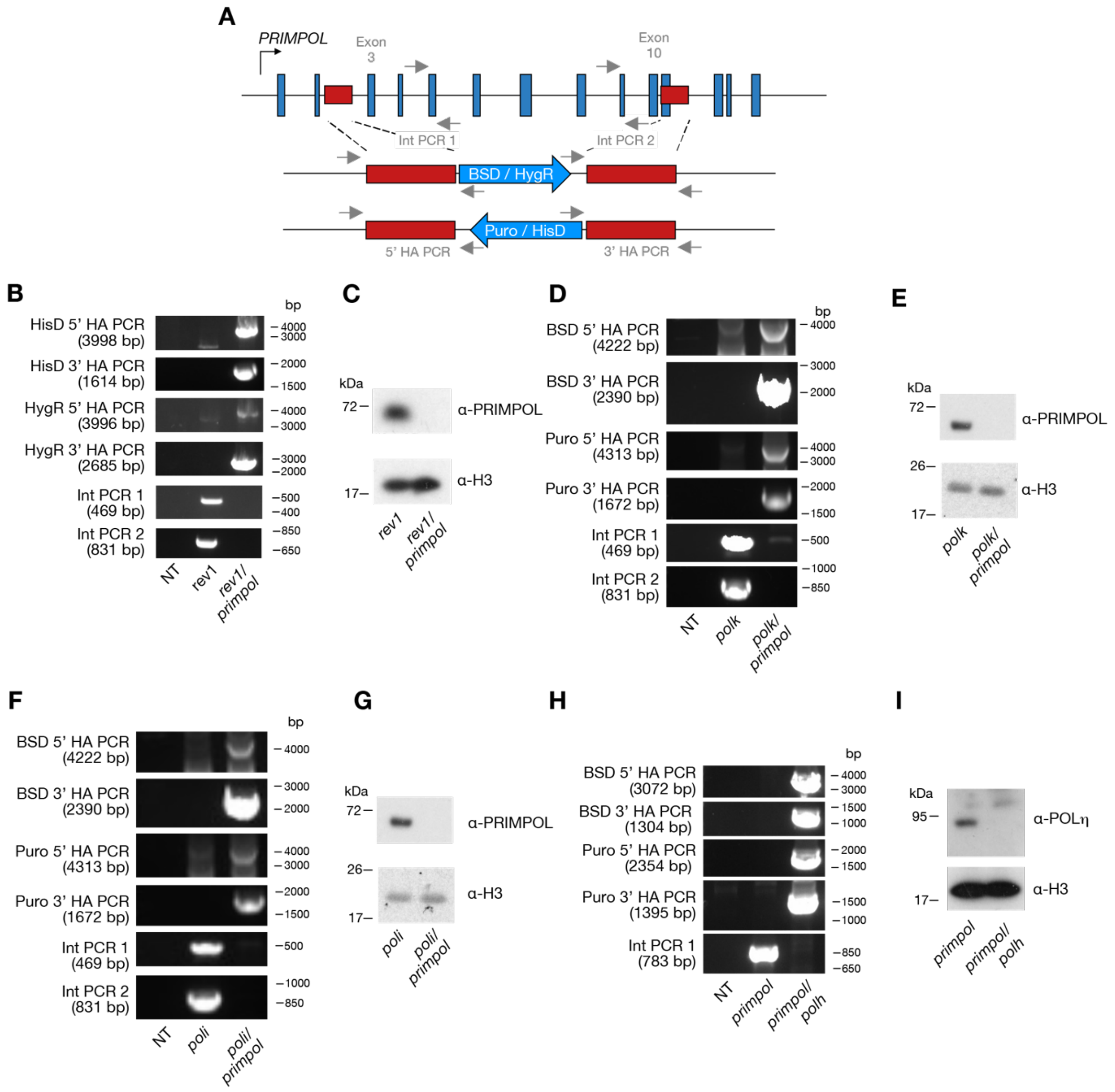

A. CRISPR/Cas9-assisted introduction of targeting constructs was used to delete a region of the *PRIMPOL* gene including the entirety of exons 3-10. The same protocol was used as in Figure S4 to generate *rev1/primpol* cells, whilst for the generation of *polk/primpol* and *poli/primpol* cells, the HisD and HygR selection cassettes were exchanged for BSD and Puro cassettes. To generate a double KO cell line lacking PRIMPOL and POLη, the *POLH* gene was targeted in TK6 *primpol* cells as described in Figure S11D. **B**. Following targeting of TK6 *rev1* cells, disruption of the *PRIMPOL* gene was confirmed by genotyping PCR. **C**. Western blot to confirm loss of PRIMPOL protein expression in the TK6 *rev1/primpol* cells. **D**. Following targeting of TK6 *polk* cells, disruption of the *PRIMPOL* gene was confirmed by genotyping PCR. **E**. Western blot to confirm loss of PRIMPOL protein expression in the TK6 *polk/primpol* cells. **F**. Following targeting of TK6 *poli* cells, disruption of the *PRIMPOL* gene was confirmed by genotyping PCR. **G**. Western blot to confirm loss of PRIMPOL protein

expression in the TK6 *poli*/*primpol* cells. **H**. Following targeting of TK6 *primpol* cells, disruption of the *POLH* gene was confirmed by genotyping PCR. **I**. Western blot to confirm loss of POLη protein expression in the TK6 *primpol*/*polh* cells. HA = homology arm.

Figure S13. PRIMPOL loss does not confer increased sensitivity to 4NQO or MMC in cells lacking a Y-family translesion synthesis polymerase.

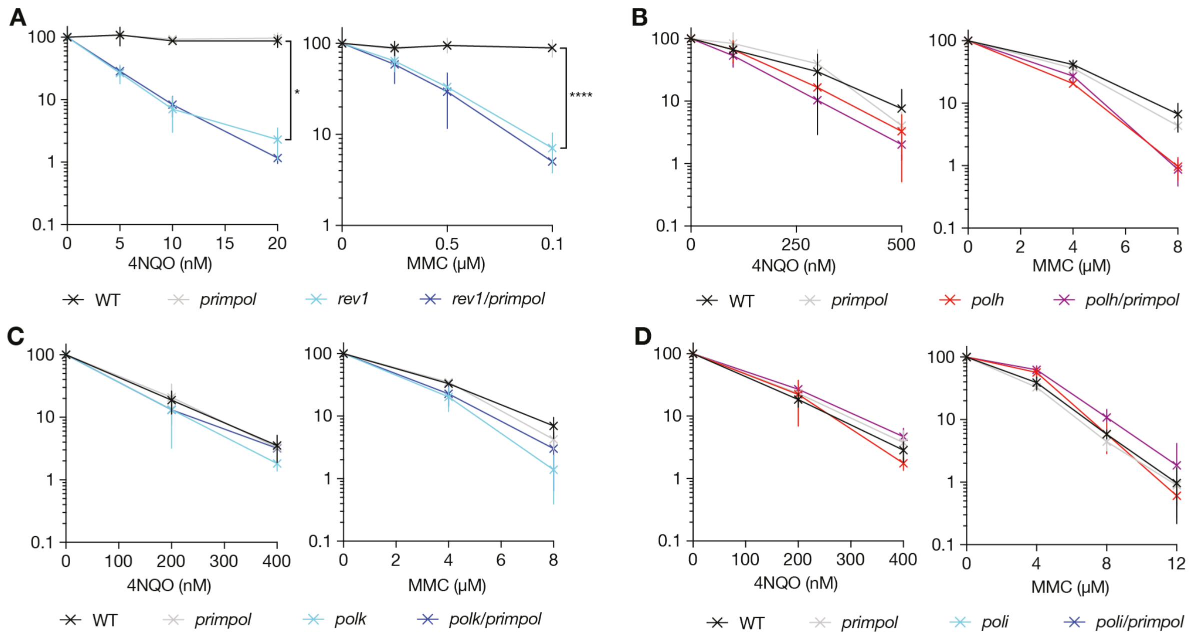

Clonogenic survival assays assessing the sensitivity of TK6 cells lacking REV1 (**A**), POLη (**B**), POLκ (**C**) or POLι (**D**) and/or PRIMPOL to 4NQO and MMC. Treatments were performed using the indicated doses for 1 hour. Results are from n = 3 biological replicates, with mean and standard deviation plotted. p-values are calculated using Brown-Forsythe and Welch ANOVA tests followed by 2-tailed unpaired t tests with Welch's correction: *: $p<0.05$; **: $p<0.005$, ***: $p<0.0005$, ****: $p<0.00005$. If not shown, there is no statistically-significant difference ($p\geq0.05$) in clonogenic survival at the highest dose of DNA damaging agent between the WT and TLS polymerase single KO, or between the TLS single KO and the double KO cell line.

Figure S14. Complementation of *rev1* and *polh* cells rescues their sensitivity to UV-C irradiation.

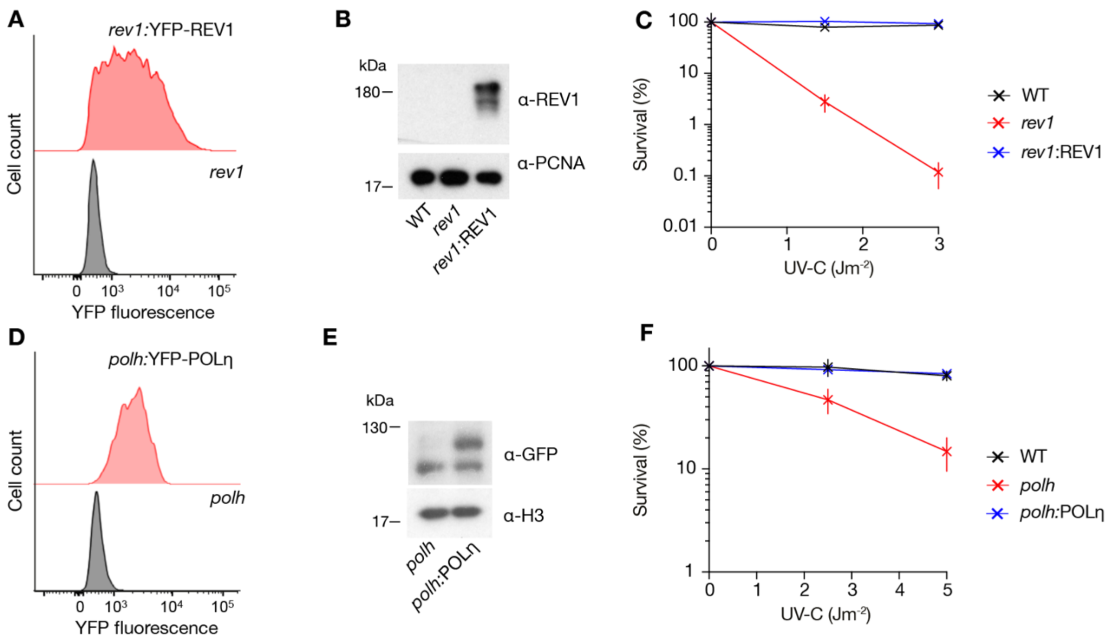

A. Complementation of TK6 *rev1* cells was achieved by transduction with a construct encoding N-terminal YFP-tagged REV1. Flow cytometry demonstrated robust YFP expression in complemented cells. **B**. Western blot showing robust expression of YFP-tagged REV1. **C**. Complementation rescues the sensitivity of TK6 *rev1* cells to UV-C irradiation, as assessed by clonogenic survival assays. Mean and standard deviation plotted, n = 3 biological replicates. **D**. Complementation of TK6 *polh* cells was achieved by transfection with a construct encoding N-terminal YFP-tagged POLη. Flow cytometry demonstrated robust YFP expression in complemented cells. **E**. Western blot showing robust expression of YFP-tagged POLη. **F**. Complementation rescues the sensitivity of TK6 *polh* cells to UV-C irradiation, as assessed by clonogenic survival assays. Mean and standard deviation plotted, n = 3 biological replicates.

S15. Complementation of *rev1/primpol* and *polh/primpol* cells rescues their sensitivity to cisplatin or UV-C irradiation respectively.

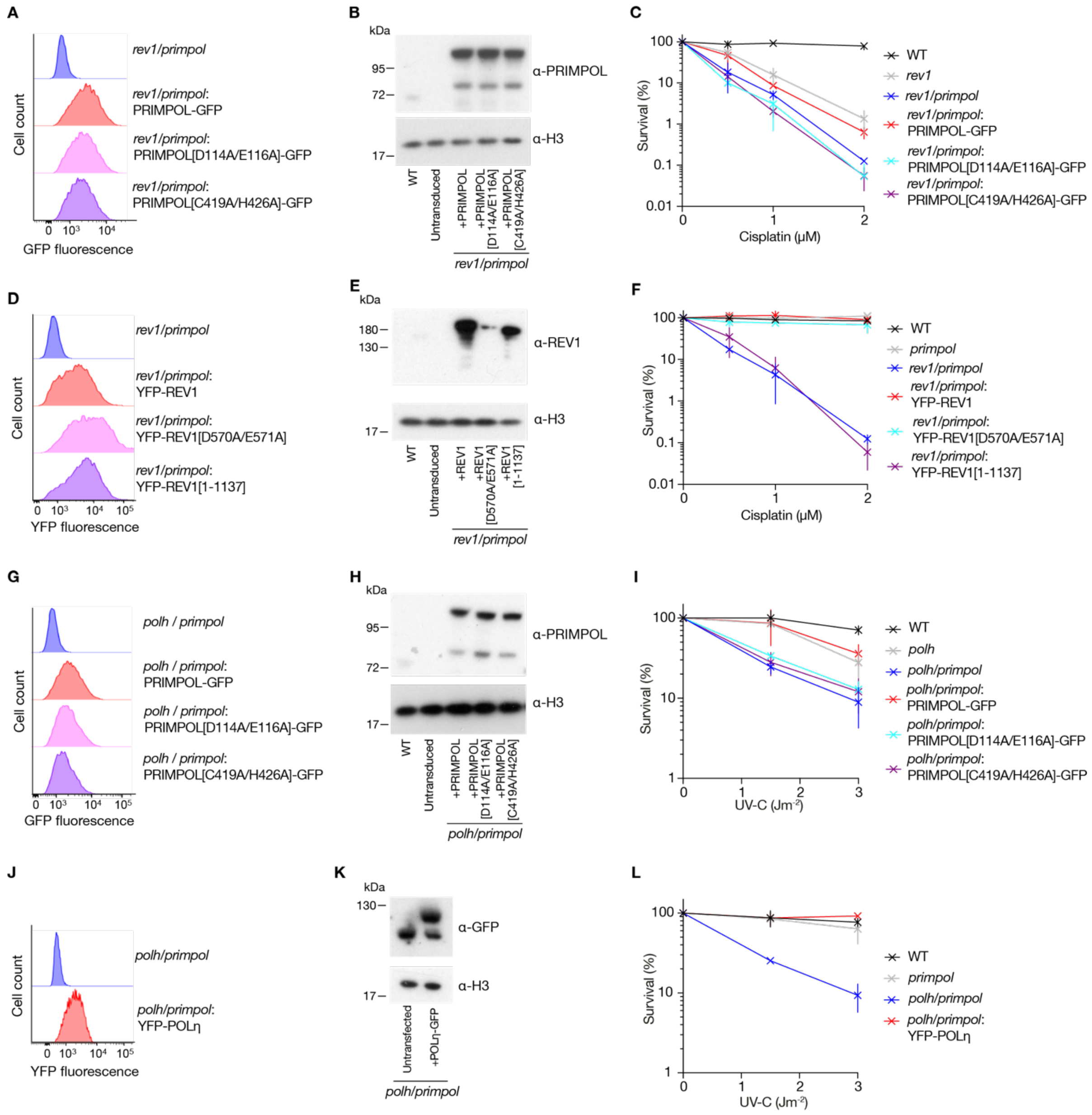

A. Complementation of TK6 *rev1/primpol* cells with GFP-tagged PRIMPOL variants. PRIMPOL[D114A/E116A] is a catalytically-dead variant, whilst the C419A H426A variant shows inhibited primase activity. Flow cytometry showing robust GFP expression in complemented *rev1/primpol* cells. **B**. Western blot showing expression of PRIMPOL-GFP variants in complemented cells. **C**. Complementation with WT PRIMPOL but not the D114A E116A or C419A H426A variants rescues the sensitivity of TK6 *rev1/primpol* to cisplatin to the level of *rev1* cells, as assessed by clonogenic survival assays. Mean and standard deviation plotted, n = 3 biological replicates. **D**. Complementation of TK6 *rev1/primpol* cells with YFP-tagged REV1 variants. REV1[D570A/E571A] is a catalytically-dead variant, whilst REV1[1-1137] encodes REV1 with a C-terminal truncation. Flow cytometry showing

robust YFP expression in complemented *rev1/primpol* cells. **E**. Western blot showing expression of YFP-REV1 variants in complemented cells. **F**. Complementation with WT REV1, the D570A E571A variant but not the C419A H426A variant rescues the sensitivity of TK6 *rev1/primpol* to cisplatin to the level of WT and *primpol* cells, as assessed by clonogenic survival assays. Mean and standard deviation plotted, n = 3 biological replicates. **G**. Complementation of TK6 *polh/primpol* cells with GFP-tagged PRIMPOL variants. Flow cytometry showing robust GFP expression in complemented *polh/primpol* cells. **H**. Western blot showing expression of PRIMPOL-GFP variants in complemented cells. **I**. Complementation with WT PRIMPOL but not the D114A E116A or C419A H426A variants rescues the sensitivity of TK6 *polh/primpol* to UV-C irradiation to the level of *polh* cells, as assessed by clonogenic survival assays. Mean and standard deviation plotted, n = 3 biological replicates. **J**. Complementation of TK6 *polh/primpol* cells with YFP-tagged POLη. Flow cytometry demonstrated robust YFP expression in complemented cells. **K**. Western blot showing expression of YFP-POLη in complemented cells. **L**. Complementation with POLη rescues the sensitivity of TK6 *polh/primpol* to UV-C irradiation to the level of WT and *primpol* cells, as assessed by clonogenic survival assays. Mean and standard deviation plotted, n = 3 biological replicates.

Figure S16: Representative image of DNA fibre staining.

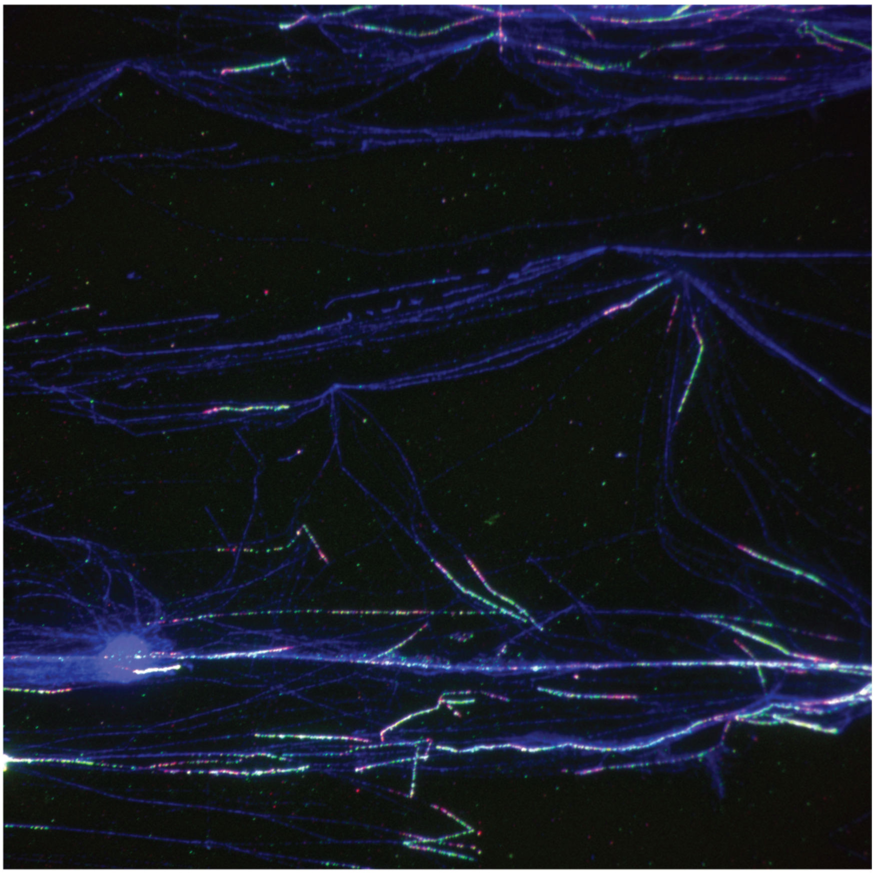

DNA replication tracts were labelled by addition of 50 µM IdU (red) for 20 minutes, followed by addition of 100 µM CldU (green) for a further 40 minutes. DNA fibres were stained using an anti-DNA antibody (blue).

Figure S17. Generation of TK6 *rad18* and *pcna*K164R mutants in wild type and *rev1* TK6 cells.

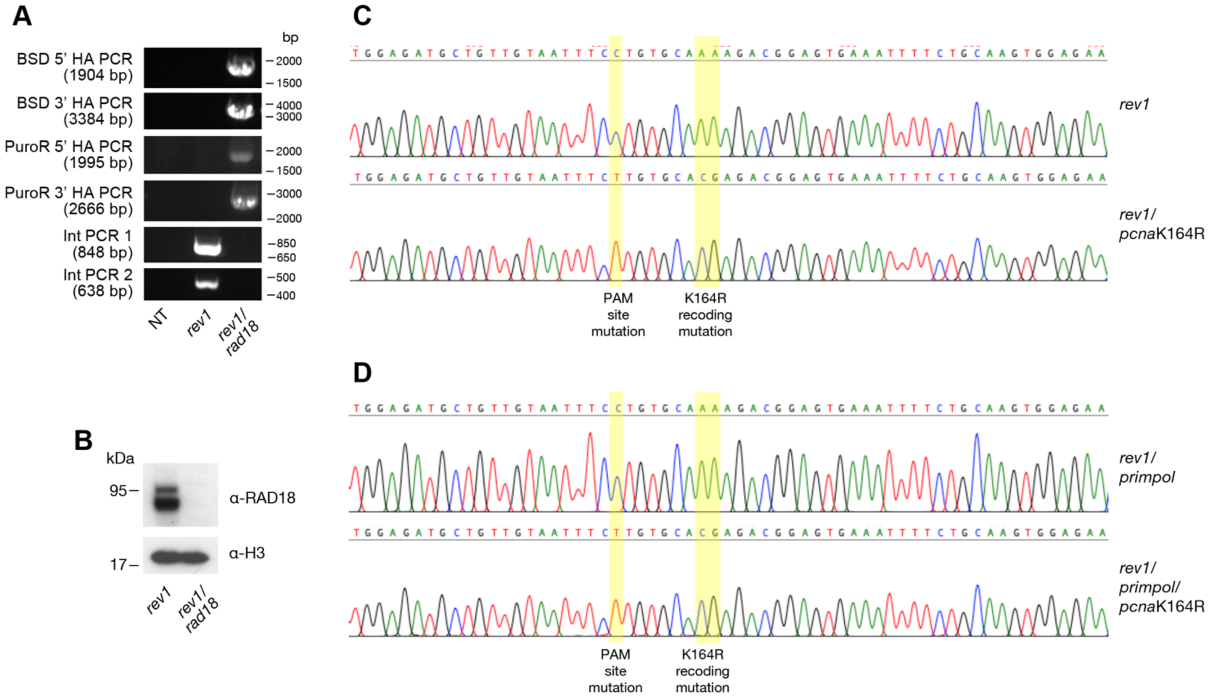

A. CRISPR/Cas9-assisted introduction of targeting constructs was used to delete a region of the *RAD18* gene including the entirety of exons 2-7. Following targeting of TK6 *rev1* cells, genotyping PCR was used to confirm insertion of targeting constructs into the *RAD18* gene. **B**. Loss of RAD18 protein expression in the TK6 *rev1*/*rad18* cells was confirmed by Western blot. **C & D**. Recoding of the *PCNA* gene to introduce the K164R point mutation was achieved by CRISPR/Cas9 targeted genome editing as described in Figure S4. Following targeting of TK6 *rev1* (C) and *rev1*/*primpol* cells (D), Sanger sequencing of a PCR amplicon confirmed the presence of the desired mutations.

Figure S18. Generation of TK6 *rev1*/*polh* cells.

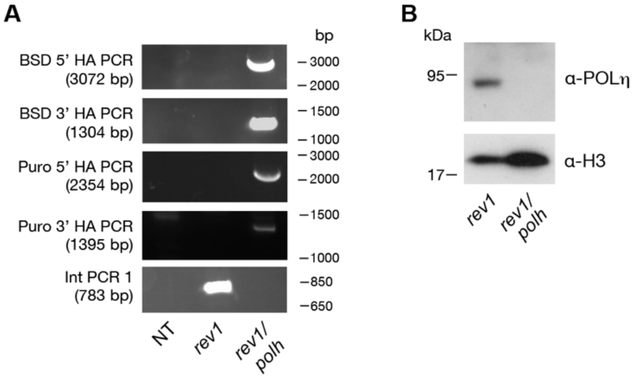

A. CRISPR/Cas9-assisted introduction of targeting constructs was used to delete a region of the *POLH* gene including the entirety of exons 2-7. The same protocol was used as in Figure S11. Following targeting of TK6 *rev1* cells, genotyping PCR was used to confirm insertion of targeting constructs into the *POLH* gene. **B**. Loss of POLη protein expression in the TK6 *rev1*/*polh* cells as confirmed by Western blot.

Supplementary Table 1. Antibodies used for Western blotting.
All antibodies were dluted in 5% milk (Marvel) dissolved in TBS, 0.1% Tween. The preferred method of transfer is indicated. For loading controls (H3, PCNA), the method of transfer chosen depended on the target of interest being visualised.

Antibody	Manufacturer (Serial number)	Dilution	Transfer method
Anti-CCDC111 (PRIMPOL)	Proteintech (29824-1-AP)	1 in 1000	iBlot2
Anti-DinB (Polκ)	Santa Cruz (sc-166667)	1 in 500	iBlot2
Anti-GFP	Abcam (ab290)	1 in 1000	iBlot2
Anti-H3	Abcam (ab1791)	1 in 25000	iBlot2 or wet transfer
Anti-HLTF	Abcam (ab183042)	1 in 1000	Wet transfer
Anti-PCNA	Santa Cruz (sc-56)	1 in 10000	iBlot2 or wet transfer
Anti-POL H (Polη)	Santa Cruz (sc-5592)	1 in 500	iBlot2
Anti-POLI (Polι)	Invitrogen (PA 5-29442)	1 in 500	Wet transfer
Anti-RAD18	Abcam (ab188235)	1 in 1000	iBlot2
Anti-REV1	Santa Cruz (sc-393022)	1 in 500	Wet transfer
Anti-SMARCAL1	Abcam (ab154226)	1 in 1000	Wet transfer
Anti-ubiquityl PCNA (Lys164)	Cell Signalling Technology (13439)	1 in 1000	iBlot2

Supplementary Table 2: Cell lines

The immediate parental cell line, the constructs used to generate the cell line (Supplementary Table 3) and the PCR primers used for genotyping (Supplementary Table 5) are all listed.

Cell line	Parental cell line	Generated using (constructs)	PCR primers for genotyping
TK6 TSCER2	N/A	N/A	N/A
TK6 TSCER2 *primpol*	TK6 TSCER2	pX458 sg*PRIMPOL* e5	*PRIMPOL* indel F + *PRIMPOL* indel R
TK6 TSCER2 *AAVS1*::Cas9-YFP-BSD	TK6 TSCER2	pBS *AAVS1*::Cas9-YFP-BSD<br>pX330 sg*AAVS1*	Ext 5' HA PCR: *AAVS1* Ext F + Gg β-actin R<br>Ext 3' HA PCR: SV40 pA F + *AAVS1* Ext R<br>Int PCR: *AAVS1* Int F + *AAVS1* Int R
TK6 TSCER2 *primpol* *AAVS1*::Cas9-YFP-BSD	TK6 TSCER2 *primpol*	pBS *AAVS1*::Cas9-YFP-BSD<br>pX330 sg*AAVS1*	Ext 5' HA PCR: *AAVS1* Ext F + Gg β-actin R<br>Ext 3' HA PCR: SV40 pA F + *AAVS1* Ext R<br>Int PCR: *AAVS1* Int F + *AAVS1* Int R
TK6 TSCE5	N/A	N/A	N/A
TK6 TSCE5 *primpol* (#2/3)	TK6 TSCE5	pBS *PRIMPOL* NeoR sense<br>pBS *PRIMPOL* HisD antisense<br>pX458 sg*PRIMPOL* U1<br>pX458 sg*PRIMPOL* U2<br>pX458 sg*PRIMPOL* D1<br>pX458 sg*PRIMPOL* D2	Ext NeoR PCR: *PRIMPOL* Ext R1 + Neo F<br>Ext HisD PCR: *PRIMPOL* Ext R1 + Gg β-actin R<br>Int PCR 1: *PRIMPOL* indel F + *PRIMPOL* indel R<br>Int PCR 2: *PRIMPOL* Int F1 + *PRIMPOL* Int R1
TK6 TSCE5 *bod1l1*	TK6 TSCE5	pBS *BOD1L1* BSD antisense<br>pBS *BOD1L1* Puro antisense<br>pX458 sg*BOD1L1* U1<br>pX458 sg*BOD1L1* U2<br>pX458 sg*BOD1L1* D1<br>pX458 sg*BOD1L1* D2	Ext BSD PCR: *BOD1L1* Ext F + BSD F<br>Ext Puro PCR: *BOD1L1* Ext F + Puro F<br>Int PCR 1: *BOD1L1* Int F1 + *BOD1L1* Int R1<br>Int PCR 2: *BOD1L1* Int F2 + *BOD1L1* Int R2<br>Int PCR 3: *BOD1L1* Int F3 + *BOD1L1* Int R3
TK6 TSCE5 *edc4*	TK6 TSCE5	pBS *EDC4* BSD sense<br>pBS *EDC4* Puro antisense<br>pX458 sg*EDC4* U1<br>pX458 sg*EDC4* U2<br>pX458 sg*EDC4* D1<br>pX458 sg*EDC4* D2	Ext BSD PCR: *EDC4* Ext F1 + Gg β-actin R<br>Ext Puro PCR: *EDC4* Ext F2 + Puro F<br>Int PCR 1: *EDC4* Int F1 + *EDC4* Int R1<br>Int PCR 2: *EDC4* Int F2 + *EDC4* Int R2
TK6 TSCE5 *paxip1*	TK6 TSCE5	pBS *PAXIP1* BSD antisense<br>pBS *PAXIP1* Puro antisense	Ext BSD PCR: *PAXIP1* Ext F + Gg β-actin R<br>Ext Puro PCR: *PAXIP1* Ext F + Puro F

		pX458 sg*PAXIP1* U1<br>pX458 sg*PAXIP1* U2<br>pX458 sg*PAXIP1* D1<br>pX458 sg*PAXIP1* D2	Int PCR 1: *PAXIP1* Int F1 + *PAXIP1* Int R1<br>Int PCR 2: *PAXIP1* Int F2 + *PAXIP1* Int R2<br>Int PCR 3: *PAXIP1* Int F3 + *PAXIP1* Int R3
TK6 TSCE5 *primpol/bod1l1*	TK6 TSCE5 *primpol* (#2/3)	pBS *BOD1L1* BSD antisense<br>pBS *BOD1L1* Puro antisense<br>pX458 sg*BOD1L1* U1<br>pX458 sg*BOD1L1* U2<br>pX458 sg*BOD1L1* D1<br>pX458 sg*BOD1L1* D2	Ext BSD PCR: *BOD1L1* Ext F + BSD F<br>Ext Puro PCR: *BOD1L1* Ext F + Puro F<br>Int PCR 1: *BOD1L1* Int F1 + *BOD1L1* Int R1<br>Int PCR 2: *BOD1L1* Int F2 + *BOD1L1* Int R2<br>Int PCR 3: *BOD1L1* Int F3 + *BOD1L1* Int R3
TK6 TSCE5 *primpol/edc4*	TK6 TSCE5 *primpol* (#2/3)	pBS *EDC4* BSD sense<br>pBS *EDC4* Puro antisense<br>pX458 sg*EDC4* U1<br>pX458 sg*EDC4* U2<br>pX458 sg*EDC4* D1<br>pX458 sg*EDC4* D2	Ext BSD PCR: *EDC4* Ext F1 + Gg β-actin R<br>Ext Puro PCR: *EDC4* Ext F2 + Puro F<br>Int PCR 1: *EDC4* Int F1 + *EDC4* Int R1<br>Int PCR 2: *EDC4* Int F2 + *EDC4* Int R2
TK6 TSCE5 *primpol/paxip1*	TK6 TSCE5 *primpol* (#2/3)	pBS *PAXIP1* BSD antisense<br>pBS *PAXIP1* Puro antisense<br>pX458 sg*PAXIP1* U1<br>pX458 sg*PAXIP1* U2<br>pX458 sg*PAXIP1* D1<br>pX458 sg*PAXIP1* D2	Ext BSD PCR: *PAXIP1* Ext F + Gg β-actin R<br>Ext Puro PCR: *PAXIP1* Ext F + Puro F<br>Int PCR 1: *PAXIP1* Int F1 + *PAXIP1* Int R1<br>Int PCR 2: *PAXIP1* Int F2 + *PAXIP1* Int R2<br>Int PCR 3: *PAXIP1* Int F3 + *PAXIP1* Int R3
TK6 TSCE5 *primpol* (#4)	TK6 TSCE5	pBS *PRIMPOL* HygR sense<br>pBS *PRIMPOL* HisD antisense v2<br>pX458 sg*PRIMPOL* U1<br>pX458 sg*PRIMPOL* U2<br>pX458 sg*PRIMPOL* D3<br>pX458 sg*PRIMPOL* D4	HisD 5' HA PCR: *PRIMPOL* Ext F + HisD F<br>HisD 3' HA PCR: *PRIMPOL* Ext R2 + Gg β-actin R<br>HygR 5' HA PCR: *PRIMPOL* Ext F + HygR R<br>HygR 3' HA PCR: *PRIMPOL* Ext R2 + HygR F<br>Int PCR 1: *PRIMPOL* Int F2 + *PRIMPOL* Int R2<br>Int PCR 2: *PRIMPOL* indel F + *PRIMPOL* indel R
TK6 TSCE5 *rev1*	TK6 TSCE5	pX458 sg*REV1* U1<br>pX458 sg*REV1* U2<br>pX458 sg*REV1* D1<br>pX458 sg*REV1* U2	Ext PCR: *REV1* Ext F + *REV1* Ext R<br>Int PCR 1: *REV1* Int F1 + *REV1* Int R1<br>Int PCR 2: *REV1* Int F2 + *REV1* Int R2<br>Int PCR 3: *REV1* Int F3 + *REV1* Int R3
TK6 TSCE5 *polh*	TK6 TSCE5	pBS *POLH* Puro sense<br>pBS *POLH* BSD antisense<br>pX458 sg*POLH* U1<br>pX458 sg*POLH* U2<br>pX458 sg*POLH* D1<br>pX458 sg*POLH* D2	BSD 5' HA PCR: *POLH* Ext F + BSD F<br>BSD 3' HA PCR: *POLH* Ext R + Gg β-actin R<br>Puro 5' HA PCR: *POLH* Ext F + Gg β-actin R<br>Puro 3' HA PCR: *POLH* Ext R + Puro F<br>Int PCR: *POLH* Int F + *POLH* Int R

TK6 TSCE5 *polk*	TK6 TSCE5	pBS *POLK* HygR sense<br>pBS *POLK* HisD antisense<br>pX458 sg*POLK* U1<br>pX458 sg*POLK* U2<br>pX458 sg*POLK* D1<br>pX458 sg*POLK* D2	HisD 5' HA PCR: *POLK* Ext F + HisD F<br>HisD 3' HA PCR: *POLK* Ext R + Gg β-actin R<br>HygR 5' HA PCR: *POLK* Ext F + HygR R<br>HygR 3' HA PCR: *POLK* Ext R + HygR F<br>Int PCR 1: *POLK* Int F1 + *POLK* Int R1<br>Int PCR 2: *POLK* Int F2 + *POLK* Int R2
TK6 TSCE5 *poli*	TK6 TSCE5	pBS *POLI* HygR sense<br>pBS *POLI* HisD antisense<br>pX458 sg*POLI* U1<br>pX458 sg*POLI* U2<br>pX458 sg*POLI* D1<br>pX458 sg*POLI* D2	HisD 5' HA PCR: *POLI* Ext F + HisD F<br>HisD 3' HA PCR: *POLI* Ext R + Gg β-actin R<br>HygR 5' HA PCR: *POLI* Ext F + HygR R<br>HygR 3' HA PCR: *POLI* Ext R + HygR F<br>Int PCR 1: *POLI* Int F1 + *POLI* Int R1<br>Int PCR 2: *POLI* Int F2 + *POLI* Int R2
TK6 TSCE5 *hltf*	TK6 TSCE5	pBS *HLTF* BSD sense<br>pBS *HLTF* Puro antisense<br>pX458 sg*HLTF* U1<br>pX458 sg*HLTF* U2<br>pX458 sg*HLTF* D1<br>pX458 sg*HLTF* D2	BSD 5' HA PCR: *HLTF* Ext F + Gg β-actin R<br>BSD 3' HA PCR: *HLTF* Ext R + BSD F<br>Puro 5' HA PCR: *HLTF* Ext F + Puro F<br>Puro 3' HA PCR: *HLTF* Ext R + Gg β-actin R<br>Int PCR 1: *HLTF* Int F1 + *HLTF* Int R1<br>Int PCR 2: *HLTF* Int F2 + *HLTF* Int R2
TK6 TSCE5 *smarcal1*	TK6 TSCE5	pBS *SMARCAL1* BSD sense<br>pBS *SMARCAL1* Puro antisense<br>pX458 sg*SMARCAL1* U1<br>pX458 sg*SMARCAL1* U2<br>pX458 sg*SMARCAL1* D	BSD 5' HA PCR: *SMARCAL1* Ext F + Gg β-actin R<br>BSD 3' HA PCR: *SMARCAL1* Ext R + BSD F<br>Puro 5' HA PCR: *SMARCAL1* Ext F + Puro F<br>Puro 3' HA PCR: *SMARCAL1* Ext R + Gg β-actin R<br>Int PCR 1: *HLTF* Int F1 + *SMARCAL1* Int R1<br>Int PCR 2: *SMARCAL1* Int F2 + *SMARCAL1* Int R2
TK6 TSCE5 *rad18*	TK6 TSCE5	pBS *RAD18* BSD sense<br>pBS *RAD18* Puro antisense<br>pX458 sg*RAD18* U1<br>pX458 sg*RAD18* U2<br>pX458 sg*RAD18* D1<br>pX458 sg*RAD18* D2	BSD 5' HA PCR: *RAD18* Ext F + Gg β-actin R<br>BSD 3' HA PCR: *RAD18* Ext R + BSD F<br>Puro 5' HA PCR: *RAD18* Ext F + Puro F<br>Puro 3' HA PCR: *RAD18* Ext R + Gg β-actin R<br>Int PCR 1: *RAD18* Int F1 + *RAD18* Int R1<br>Int PCR 2: *RAD18* Int F2 + *RAD18* Int R2
TK6 TSCE5 *pcna*K164R	TK6 TSCE5	N/A	*PCNA* e5 F + *PCNA* e5 R
TK6 TSCE5 *primpol/polh*	TK6 TSCE5 *primpol* (#4)	pBS *POLH* Puro sense<br>pBS *POLH* BSD antisense<br>pX458 sg*POLH* U1<br>pX458 sg*POLH* U2<br>pX458 sg*POLH* D1<br>pX458 sg*POLH* D2	BSD 5' HA PCR: *POLH* Ext F + BSD F<br>BSD 3' HA PCR: *POLH* Ext R + Gg β-actin R<br>Puro 5' HA PCR: *POLH* Ext F + Gg β-actin R<br>Puro 3' HA PCR: *POLH* Ext R + Puro F<br>Int PCR: *POLH* Int F + *POLH* Int R

TK6 TSCE5 *primpol/hltf*	TK6 TSCE5 *primpol* (#4)	pBS *HLTF* BSD sense<br>pBS *HLTF* Puro antisense<br>pX458 sg*HLTF* U1<br>pX458 sg*HLTF* U2<br>pX458 sg*HLTF* D1<br>pX458 sg*HLTF* D2	BSD 5' HA PCR: *HLTF* Ext F + Gg β-actin R<br>BSD 3' HA PCR: *HLTF* Ext R + BSD F<br>Puro 5' HA PCR: *HLTF* Ext F + Puro F<br>Puro 3' HA PCR: *HLTF* Ext R + Gg β-actin R<br>Int PCR 1: *HLTF* Int F1 + *HLTF* Int R1<br>Int PCR 2: *HLTF* Int F2 + *HLTF* Int R2
TK6 TSCE5 *primpol/smarcal1*	TK6 TSCE5 *primpol* (#4)	pBS *SMARCAL1* BSD sense<br>pBS *SMARCAL1* Puro antisense<br>pX458 sg*SMARCAL1* U1<br>pX458 sg*SMARCAL1* U2<br>pX458 sg*SMARCAL1* D	BSD 5' HA PCR: *SMARCAL1* Ext F + Gg β-actin R<br>BSD 3' HA PCR: *SMARCAL1* Ext R + BSD F<br>Puro 5' HA PCR: *SMARCAL1* Ext F + Puro F<br>Puro 3' HA PCR: *SMARCAL1* Ext R + Gg β-actin R<br>Int PCR 1: *HLTF* Int F1 + *SMARCAL1* Int R1<br>Int PCR 2: *SMARCAL1* Int F2 + *SMARCAL1* Int R2
TK6 TSCE5 *rev1/primpol*	TK6 TSCE5 *rev1*	pBS *PRIMPOL* HygR sense<br>pBS *PRIMPOL* HisD antisense v2<br>pX458 sg*PRIMPOL* U1<br>pX458 sg*PRIMPOL* U2<br>pX458 sg*PRIMPOL* D3<br>pX458 sg*PRIMPOL* D4	HisD 5' HA PCR: *PRIMPOL* Ext F + HisD F<br>HisD 3' HA PCR: *PRIMPOL* Ext R + Gg β-actin R<br>HygR 5' HA PCR: *PRIMPOL* Ext F + HygR R<br>HygR 3' HA PCR: *PRIMPOL* Ext R + HygR F<br>Int PCR 1: *PRIMPOL* Int F + *PRIMPOL* Int R<br>Int PCR 2: *PRIMPOL* indel F + *PRIMPOL* indel R
TK6 TSCE5 *rev1/rad18*	TK6 TSCE5 *rev1*	pBS *RAD18* BSD sense<br>pBS *RAD18* Puro antisense<br>pX458 sg*RAD18* U1<br>pX458 sg*RAD18* U2<br>pX458 sg*RAD18* D1<br>pX458 sg*RAD18* D2	BSD 5' HA PCR: *RAD18* Ext F + Gg β-actin R<br>BSD 3' HA PCR: *RAD18* Ext R + BSD F<br>Puro 5' HA PCR: *RAD18* Ext F + Puro F<br>Puro 3' HA PCR: *RAD18* Ext R + Gg β-actin R<br>Int PCR 1: *RAD18* Int F1 + *RAD18* Int R1<br>Int PCR 2: *RAD18* Int F2 + *RAD18* Int R2
TK6 TSCE5 *rev1/pcna*K164R	TK6 TSCE5 *rev1*	N/A	*PCNA* e5 F + *PCNA* e5 R
TK6 TSCE5 *rev1/polh*	TK6 TSCE5 *rev1*	pBS *POLH* Puro sense<br>pBS *POLH* BSD antisense<br>pX458 sg*POLH* U1<br>pX458 sg*POLH* U2<br>pX458 sg*POLH* D1<br>pX458 sg*POLH* D2	BSD 5' HA PCR: *POLH* Ext F + BSD F<br>BSD 3' HA PCR: *POLH* Ext R + Gg β-actin R<br>Puro 5' HA PCR: *POLH* Ext F + Gg β-actin R<br>Puro 3' HA PCR: *POLH* Ext R + Puro F<br>Int PCR: *POLH* Int F + *POLH* Int R
TK6 *polk/primpol*	TK6 *polk*	pBS *PRIMPOL* BSD sense<br>pBS *PRIMPOL* Puro antisense<br>pX458 sg*PRIMPOL* U1<br>pX458 sg*PRIMPOL* U2<br>pX458 sg*PRIMPOL* D3<br>pX458 sg*PRIMPOL* D4	BSD 5' HA PCR: *PRIMPOL* Ext F + Gg β-actin R<br>BSD 3' HA PCR: *PRIMPOL* Ext R2 + BSD F<br>Puro 5' HA PCR: *PRIMPOL* Ext F + Puro F<br>Puro 3' HA PCR: *PRIMPOL* Ext R2 + Gg β-actin R<br>Int PCR 1: *PRIMPOL* Int F2 + *PRIMPOL* Int R2<br>Int PCR 2: *PRIMPOL* indel F + *PRIMPOL* indel R

TK6 *poli/primpol*	TK6 *poli*	pBS *PRIMPOL* BSD sense<br>pBS *PRIMPOL* Puro antisense<br>pX458 sg*PRIMPOL* U1<br>pX458 sg*PRIMPOL* U2<br>pX458 sg*PRIMPOL* D3<br>pX458 sg*PRIMPOL* D4	BSD 5' HA PCR: *PRIMPOL* Ext F + Gg β-actin R<br>BSD 3' HA PCR: *PRIMPOL* Ext R2 + BSD F<br>Puro 5' HA PCR: *PRIMPOL* Ext F + Puro F<br>Puro 3' HA PCR: *PRIMPOL* Ext R2 + Gg β-actin R<br>Int PCR 1: *PRIMPOL* Int F2 + *PRIMPOL* Int R2<br>Int PCR 2: *PRIMPOL* indel F + *PRIMPOL* indel R
TK6 TSCE5 *rad18/primpol*	TK6 TSCE5<br>*rad18*	pBS *PRIMPOL* HygR sense<br>pBS *PRIMPOL* HisD antisense v2<br>pX458 sg*PRIMPOL* U1<br>pX458 sg*PRIMPOL* U2<br>pX458 sg*PRIMPOL* D3<br>pX458 sg*PRIMPOL* D4	HisD 5' HA PCR: *PRIMPOL* Ext F + HisD F<br>HisD 3' HA PCR: *PRIMPOL* Ext R2 + Gg β-actin R<br>HygR 5' HA PCR: *PRIMPOL* Ext F + HygR R<br>HygR 3' HA PCR: *PRIMPOL* Ext R2 + HygR F<br>Int PCR 1: *PRIMPOL* Int F2 + *PRIMPOL* Int R2<br>Int PCR 2: *PRIMPOL* indel F + *PRIMPOL* indel R
TK6 TSCE5<br>*pcna*K164R/*primpol*	TK6 TSCE5<br>*pcna*K164R	pBS *PRIMPOL* HygR sense<br>pBS *PRIMPOL* HisD antisense v2<br>pX458 sg*PRIMPOL* U1<br>pX458 sg*PRIMPOL* U2<br>pX458 sg*PRIMPOL* D3<br>pX458 sg*PRIMPOL* D4	HisD 5' HA PCR: *PRIMPOL* Ext F + HisD F<br>HisD 3' HA PCR: *PRIMPOL* Ext R2 + Gg β-actin R<br>HygR 5' HA PCR: *PRIMPOL* Ext F + HygR R<br>HygR 3' HA PCR: *PRIMPOL* Ext R2 + HygR F<br>Int PCR 1: *PRIMPOL* Int F2 + *PRIMPOL* Int R2<br>Int PCR 2: *PRIMPOL* indel F + *PRIMPOL* indel R
TK6 TSCE5 *rev1*/<br>*pcna*K164R/*primpol*	TK6 TSCE5 *rev1*/*primpol*	N/A	*PCNA* e5 F + *PCNA* e5 R
HEK293ET	N/A	N/A	N/A

Supplementary Table 3: Constructs used for generating cell lines

For sgRNA delivery constructs, the sgRNA sequences listed were cloned into the BbsI sites of pX458 (Ran et al., 2013), pX330 (Cong et al., 2013) and pKLV2-U6gRNA5(BbsI)-PGKpuro2A-BFP-W (pKLV2 U6-Empty) (Tzelepis et al., 2016). Targeting constructs were produced using Gibson assembly, using pBluescript SK+ (pBS) as a backbone. Homology arms (HA) spanning the specified regions of the genome were amplified by primers binding to the terminal sites indicated. Gibson assembly overlaps were added to the 5' ends of the following primers:

5' HA forward primer: AATTGGGTACCGGGCCCCCCCTCGA

5' HA reverse primer: CAGCCAAGCTGGGCGTAATCATGGTCGAC**GGATCC**

3' HA forward primer: AGTGAAGCAGAACGTGGGGCTCACCTCGA**GGATCC**

3' HA reverse primer: TGGAGCTCCACCGCGGTGGCGGCCGC

These overlaps allowed construction of a targeting construct with an antisense HisD cassette. BamHI sites are highlighted in bold. The HisD selection cassette could then be exchanged with a HygR, BSD or Puro cassette by BamHI digestion to remove the HisD cassette followed by ligation to introduce the desired cassette as a BamHI fragment. For the *POLI* targeting constructs, the BamHI sites within the pBS *POLI* HisD antisense construct were replaced with BglII sites to prevent interference due to a BamHI site within the 5' HA.

Construct	sgRNA sequence	5' HA	3' HA
pX458 sg*PRIMPOL* e5	GTTTAACAAACCTGCCAACCC		
pBS *AAVS1*::Cas9-YFP-BSD		TGCTTTCTCTGACCAGCATTCTCTCCCCTGGG…CCCCTCCACCCCACAGTGGGGC	ACTAGGGACAGGATTGGTGACAGAAAAGCCCC…CCCTACAGGGGTTCCTGGCTCTGCTCT
pX330 sg*AAVS1*	GGGGCCACTAGGGACAGGAT		
pBS *PRIMPOL* HisD antisense v1		CCCGGCTGAAGCAGTTTTTA…GGCTGGGCAGTTTGATAGGT	TCAGAAGGTAACAGCATGAGAC…ACACAAACCTTAGGCTCAGGA
pBS *PRIMPOL* NeoR sense			
pX458 sg*PRIMPOL* U1	CGTTGAATTCAGATGACGGC		
pX458 sg*PRIMPOL* U2	TGACGGCTGGATGTCCGGAA		
pX458 sg*PRIMPOL* D1	CTCATACCTGGGACCGATTA		
pX458 sg*PRIMPOL* D2	TTGACACCTTAATCGGTCCC		

pBS *BOD1L1* BSD antisense			
pBS *BOD1L1* Puro antisense			
pX458 sg*BOD1L1* U1	CGCCCGGCGACGGTCGAAAC		
pX458 sg*BOD1L1* U2	CTTCGACCCGCGCGACACCT		
pX458 sg*BOD1L1* D1	CGTGTTCCTCCATAAGCCTA		
pX458 sg*BOD1L1* D2	GTAGGCAGCCTGGCGTCTGA		
pBS *EDC4* BSD sense		TTTGGTAGAGCCTGGCATGG...ACTCTGAAAGGGGAGAGGCT	TTCAGTTCCTGCAGGCTGAGCC...GGCAGGACTTATAGACCAGTCGTA
pBS *EDC4* Puro antisense			
pX458 sg*EDC4* U1	GGCCATCCAGTGCCTACAAT		
pX458 sg*EDC4* U2	TGTAGGCACTGGATGGCCGT		
pX458 sg*EDC4* D1	GGACCACATGGGCTCCGTTA		
pX458 sg*EDC4* D2	CCCATGTGGTCCCGAGTGAT		
pBS *PAXIP1* BSD antisense		TGTGCTGAGGAATGAAGGCA...GCCCTGCATATACCCCAGAA	CCTTTCCTGTTTTGCAAGCCA...GGTGCAGAAACGCTCTTACC
pBS *PAXIP1* Puro antisense			
pX458 sg*PAXIP1* U1	ATACAGAGCAGACCGTAAAT		
pX458 sg*PAXIP1* U2	GCTCCCTCTTCCTCGATGTA		
pX458 sg*PAXIP1* D1	AATGCAGAGTTCGTTCTGAC		
pX458 sg*PAXIP1* D2	TGGAGTGCTCACTCAAACGC		
pBS *PRIMPOL* HisD antisense v2		CCCGGCTGAAGCAGTTTTTA...GGCTGGGCAGTTTGATAGGT	GCATTTTAGGAATTCGGCGT...GGGAATGATCGGGAAGCTCT
pBS *PRIMPOL* HygR sense			
pBS *PRIMPOL* BSD sense			
pBS *PRIMPOL* Puro antisense			
pX458 sg*PRIMPOL* D3	TATCATATAACAGTTAACAA		
pX458 sg*PRIMPOL* D4	AATAAAGATGGCATTAAAGG		

pX458 sg*REV1* U1	CCATTTGCTTGCGCAGAATC		
pX458 sg*REV1* U2	TTCTCCCACAATGATGAATC		
pX458 sg*REV1* D1	ATGACCACTGTCACAGACAA		
pX458 sg*REV1* D2	CACAGACAATGGCAGTCCAC		
pBS *POLH* Puro sense		AACGGAAAACCTCAACAGCTTTA…CCCAGGTCATACCCATACCAATT	CAGTGGCTTGCTTGGCATTT…GGTACAATGGTGGCTGTTGC
pBS *POLH* BSD antisense			
pX458 sg*POLH* U1	TATTTAAAGCAGCCTGGCGG		
pX458 sg*POLH* U2	TAAGTTAGTATATATAGTTG		
pX458 sg*POLH* D1	TTGCTAGGGCATTAAACACA		
pX458 sg*POLH* D2	AAATGTGTGTGTGCTTGCTA		
pBS *POLK* HygR sense		GAACAGCCTGAGGAAGGCAT…TGTGCCTAAGGGTATGGAGAGA	TGGAAATCAATCCTGTTAAGAGATAATTC…GCCAATCCAATCCCATGACTGA
pBS *POLK* HisD antisense			
pX458 sg*POLK* U1	GGAGAGATGAAAGGGTTAGT		
pX458 sg*POLK* U2	AGGGAATTATATTTGAGGAT		
pX458 sg*POLK* D1	TACTTCCATTATACATCAAT		
pX458 sg*POLK* D2	GATTTCCAATTGATGTATAA		
pBS *POLI* HygR sense		TCTTTACCTTGTACCGCCCA…AAATGTAAGGGCATGCTGCT	GTTGTAAAGATGGTAATAGGGGGT…CACCACGGGCCCTTATTTCA
pBS *POLI* HisD antisense			
pX458 sg*POLI* U1	GCTCTTAGTGTAAACCAGAA		
pX458 sg*POLI* U2	AAGAGCTACTATAAACTTCC		
pX458 sg*POLI* D1	CTTGGAACTGACAGATTATC		
pX458 sg*POLI* D2	TTCATTCTATATACGGTATG		
pBS *HLTF* BSD sense		TTAAAGGCTACAGGATTGCTGG…ACAACGAGATCCTAATAACCCTTAT	AGTAAGTAGTTCTGTAAATT…TGTGGTTGTGTAGTACAGGCAT
pBS *HLTF* Puro antisense			
pX458 sg*HLTF* U1	AATGTGAATGGAAATCAAGT		

pX458 sg*HLTF* U2	CAATTTGTTGTCCATGATAT		
pX458 sg*HLTF* D1	ATGCTTCTGTCCTTAAAAGC		
pX458 sg*HLTF* D2	CTTCTGTCCTTAAAAGCAGG		
pBS *SMARCAL1* BSD sense		GCCAAATTGTTTTCCAGCAGGT…CTTGACCATGTCCCTTCCCC	GCTGCAGCCAAGGAAATGAC…CTGGAGTAAGTGAGCAGGCA
pBS *SMARCAL1* Puro antisense			
pX458 sg*SMARCAL1* U1	TAAAGCAGGGGACTATGATG		
pX458 sg*SMARCAL1* U2	GTGGCTTTGAAATACAGGCA		
pX458 sg*SMARCAL1* D	ATCCAGGGCAGCTCTGGTCC		
pBS *RAD18* BSD sense		TTACTGGTTCACTCAGGCCAC…CGGGCTGCTGACGTAATG	AGTGAGTTATGCTTACTTTCTGGAT…CTGCCCTCATTTGTGCAAC
pBS *RAD18* Puro antisense			
pX458 sg*RAD18* U1	GCTGACGTAATGCGGTAGCG		
pX458 sg*RAD18* U2	GTAGCGCGGGGAATTTCGAG		
pX458 sg*RAD18* D1	TAGGATGCAAAGCATCGCAT		
pX458 sg*RAD18* D2	GATGCTTTGCATCCTAAATC		
pKLV2-U6gRNA5(BbsI)-PGKpuro2A-BFP-W (pKLV2 U6-Empty)	N/A		
pKLV2 U6-*sgB2M* 1	CACGTCATCCAGCAGAGAA		
pKLV2 U6-*sgB2M* 2	TCCTGAATTGCTATGTGTC		
pKLV2 U6-*sgB2M* 3	AGTCAACTTCAATGTCGGA		
pKLV2 U6-*sgB2M* 4	GGAGAGAGAATTGAAAAAG		
pKLV2 U6-*sgB2M* 5	CTTGTCTTTCAGCAAGGAC		

Supplementary Table 4. Constructs used for complementation.

The construct M6P N-terminal GFP IRES BSD, derived from M5P (1) was used as a base for producing complementation constructs for gamma retroviral transduction.

<table>
<tr><th>Construct</th><th>Source</th></tr>
<tr><td>pXPSN-neo-YFP-hRAD18</td><td>(2)</td></tr>
<tr><td>pXPSN-neo-YFP-hPolη</td><td>The human POLH open reading frame (SalI - NotI) was cloned in a three-way ligation with enhanced yellow fluorescent protein (eYFP; Clontech) (HindIII - SalI) into pXPSN cut with HindIII – NotI (3). This fuses YFP to the N-terminus of RAD18. The reading frame of the fusion is as described for REV1 in (3). The β-actin promoter-YFP-RAD18 cassettes were subsequently cloned into pLoxNeo (4).</td></tr>
<tr><td>M6P PRIMPOL-GFP IRES BSD</td><td rowspan="3">PRIMPOL coding sequences were amplified from the constructs pXPSN-BSR-PRIMPOL-YFP, pXPSN-PRIMPOL D114A E116A-YFP and pXPSN-PRIMPOL C419A H426A-YFP (5) using primers with the sequences ATACGTCTCACATGAATAGAAAATGGGAAGCAAAAC and ATACGTCTCACATGATCTCTTGTAATACTTCTATAA. Following BsmI-v2 digestion, these PCR products were ligated into NcoI-digested M6P N-terminal GFP IRES BSD, screened for the correct orientation insertion, and the desired sequence confirmed by Sanger sequencing.</td></tr>
<tr><td>M6P PRIMPOL E114A D116A-GFP IRES BSD</td></tr>
<tr><td>M6P PRIMPOL C419A H426A-GFP IRES BSD</td></tr>
<tr><td>m6P PRIMPOL-GFP IRES NeoR</td><td rowspan="3">To replace the BSD sequence with a sequence conferring G418 resistance, M6P PRIMPOL-GFP IRES BSD constructs were digested with BmgBI and HpaI. The NeoR sequence was amplified from a NeoR resistance cassette using the primers CGTCTAGGCCCCCCGAACCACGGGGACGTGGTTTTCCTTTGAAAAACACGATGATAATATGGGATCGGCCATTGAACAAG and ACCACTGATATCCTGTCTTTAACAAATTGGACTAATCCGGATCTGTTAACTACCTGCCTACGGCCGCTCAGAAGAACTCGTCAAGAAG. The NeoR cassette was inserted into the digested backbones using Gibson assembly.</td></tr>
<tr><td>M6P PRIMPOL E114A D116A-GFP IRES NeoR</td></tr>
<tr><td>M6P PRIMPOL C419A H426A-GFP IRES NeoR</td></tr>
<tr><td>M6P YFP-REV1</td><td rowspan="3">REV1 and REV1 D570A E571A sequences were amplified from the constructs pXPSN-BSR-YFP-REV1 and pXPSN-BSR-YFP-REV1[D570A E571] (3) using primers with the sequences ATACGTCTCACATGGTGAGCAAGGGCGAGGA and ATACGTCTCCGGCCGCTCAGGGGCAGCAGCTTTATGTAACTTTTAATGTGC whilst the REV1[1-1137] sequence was amplified from pXPSN-BSR-YFP-REV1[1-1137] (6) using primers with the sequences ATACGTCTCACATGGTGAGCAAGGGCGAGGA and ATACGTCTCCGGCCGCTCAGGGGCAGCAGCTTTAACCTGAAGTAGAAGCAG. Following BsmI-v2 digestion, these PCR products were ligated into NcoI/NotI-digested M6P N-terminal GFP IRES BSD and the sequence confirmed by Sanger sequencing.</td></tr>
<tr><td>M6P YFP-REV1 D570A E571A</td></tr>
<tr><td>M6P YFP-REV1 1-1137</td></tr>
</table>

Supplementary Table 5. Oligonucleotides

**Oligonucleotide**	**Sequence**
Gg β-actin R	CCGCCTCGCCATAAAAGGAA
SV40 pA F	CTCCCCCTGAACCTGAAACATA
HisD F	GTATTGGTCACCACGGCC
HygR F	CGGGGATTCCCAATACGAGG
HygR R	ACAAAATTGGGGGTGGGGAG
BSD F	CGACCTCGCGGAGTTCTAC
Puro F	CAGCGCCCGACCGAAAGGAGCGCACGACC
NeoR F	GACCGCTATCAGGACATAGCG
PRIMPOL indel F	TGAGCCATAAACCTATGGTGAA
PRIMPOL indel R	CCAGACAGCAAAATTGCAAAC
AAVS1 Ext F	CGGAACTCTGCCCTCTAACG
AAVS1 Ext R	TGGGATACCCCGAAGAGTGA
AAVS1 Int F	TCGGGTCACCTCTCACTCCTTT
AAVS1 Int R	CTGGCTCCATCGTAAGCAAACC
PRIMPOL Ext F	AAAAGCAACGTGGGGCATTC
PRIMPOL Ext R1	GTTGCTTCTGGATTTTTACTGCAGAC
PRIMPOL Ext R2	AAGAACAGAAGAAACGTCAACTGAT
PRIMPOL Int F1	TTCTTGGAACAGCATGCAGC
PRIMPOL Int R1	GCCACTGTGCCTGACCTAAT
PRIMPOL Int F2	TCATGTTGGTAAGTACACGGCT
PRIMPOL Int R2	ACAGGTCCAAAGCTAAGGGAAC
BOD1L1 Ext F	GTCTAGTTTGACAATGAAAATGGGCTC
BOD1L1 Int F1	GGTGCGAGGTCAGGGGTTCTTGT
BOD1L1 Int R1	GGAAGCACCTTGTCGCCTTGTG
BOD1L1 Int F2	CTCACTGCCAGCAGAAGTGA
BOD1L1 Int R2	ACAGAGGGATCCTTAGCCGT
BOD1L1 Int F3	CAGTGGCTTGAGACTGAGCA
BOD1L1 Int R3	TGGTGGGTATACGTCAGGGT
EDC4 Ext F1	CATGAGTTGGGCGTGGAC
EDC4 Ext F2	CTGCTGCTCAGCTGCTTTG
EDC4 Int F1	CCTGCATTGGGATTTTGGCC
EDC4 Int R1	AAGCTTGGTTTTCCCACCCA
EDC4 Int F2	TTTAGCACAGGTACAGCGCA
EDC4 Int R2	TTCAGGTCAGCAGCTGTCAG
PAXIP1 Ext F	GTAGACACCACTGAAATGTCACTCAG
PAXIP1 Int F1	TCACTGCCTGCCTTTCTCAG
PAXIP1 Int R1	TTGACACTGCTCCCCTTTCC
PAXIP1 Int F2	TTGAGTCAGACGTCACAGGC
PAXIP1 Int R2	TCCGCAATTGCAAACACACA
PAXIP1 Int F3	CTGTTCTCGAACTCCTGGCA
PAXIP1 Int R3	CCTCCAGTGGCAATGTCCAA

POLH Ext F	AACCCAGCATTTTCGGCAAC
POLH Ext R	AAAGCCCACCCCAGGAATAAA
POLH Int F	ATCGAGTGGTTGCTCTCGTG
POLH Int R	GGACTCACGAACTTGTGCCA
POLK Ext F	AGCAAAAGGAACAACGTGCAT
POLK Ext R	CTGGGCACAGAAAACAAAAGTGA
POLK Int F1	GAGGCTGTGCCCACAACTTAT
POLK Int R1	GCACTGCATGATAGACCAGTT
POLK Int F2	CCATAGCAAACCCTGGGGAG
POLK Int R2	ACTGCTTTGCAGCTACTCCT
POLI Ext F	TCCCTCTGCCTTGTGTTACG
POLI Ext R	CTCGATGGCTGCTTAACTAGGA
POLI Int F1	ACCTGCAACTATGAAGCTAGGAAA
POLI Int R1	GCCAGTGCACACTCATTTTCATT
POLI Int F2	ACGTCAATTAACTGAGGGCTTT
POLI Int R2	TCACACAGCGAACTCACTGAA
HLTF Ext F	GATGGTCTTGATCTCTTGACCTC
HLTF Ext R	CAACAAGGTAGCCTCACCCA
HLTF Int F1	GAACCAGCTGAGGTATTGAACC
HLTF Int R1	TGGACATTTTCTGGTCGGTCC
HLTF Int F2	GGGTCTTTTGCTTAGACTGCG
HLTF Int R2	TGCCAGTAGGGAAAGTCTCTCA
SMARCAL1 Ext F	GCTGCTAGCTCCGTCTAGTG
SMARCAL1 Ext R	GACCACCAAGATTGATTTTAGTGAC
SMARCAL1 Int F1	GTCCCAGAGGCAGACCTTTC
SMARCAL1 Int R1	ACGGCATTTTGCTTCTCCCA
SMARCAL1 Int F2	TCATCGCAGTCAAGCCAACT
SMARCAL1 Int R2	ACCTCAGTGCCAGCATCTTC
RAD18 Ext F	TGGGAATGTGGGCTTAGTCG
RAD18 Ext R	GCACATACAAAAAGCGACAAGC
RAD18 Int F1	TGCTCTATGGGTCCTGTGGA
RAD18 Int R1	CACCCCACTCACACCTTTGT
RAD18 Int F2	AGCAGGGGAGCAGGTTAATG
RAD18 Int R2	GCGGGATGACTAGGTTAGGC
PCNA e5 F	TGAGTTGTTTAGGTGTTGCCTTT
PCNA e5 R	CAAGGGCTCTCTAGGTACTCTC
PCNA K164R seq	GCCTTAGTGAGAAGTCTCCTTCC
REV1 Ext F	CACTGGGTAATCTGCTTGGGAT
REV1 Ext R	GGCAGCAAATACCTCAGGGT
REV1 Int F1	TGTTCTCGCTCTAGGCAACT
REV1 Int R1	GCTTTCCATTCACAACGCAGA

REV1 Int F2	AGCAAAACCAGATGGGCAGT
REV1 Int R2	ATCCTGCCCCTTAGGTGAGA
REV1 Int F3	ACTGGCATGAAGGGTAAACGTC
REV1 Int R3	AATGCCGTCAAACCAACCTCC
gRNA-HiSeq-SE50 F1	ACACTCTTTCCCTACACGACGCTCTTCCGATCTCTTGTGGAAAGGACGAAACA
gRNA-HiSeq-SE50 R1	GACTGGAGTTCAGACGTGTGCTCTTCCGATCTCCGACTCGGTGCCACTTTTTCAA
P5 primer	AATGATACGGCGACCACCGAGATCTACACTCTTTCCCTACACGAC
P7 Index 1	CAAGCAGAAGACGGCATACGAGAT**CGTGAT**GTGACTGGAGTTCAGACGT
P7 Index 2	CAAGCAGAAGACGGCATACGAGAT**ACATCG**GTGACTGGAGTTCAGACGT
P7 Index 3	CAAGCAGAAGACGGCATACGAGAT**GCCTAA**GTGACTGGAGTTCAGACGT
P7 Index 4	CAAGCAGAAGACGGCATACGAGAT**TGGTCA**GTGACTGGAGTTCAGACGT
P7 Index 5	CAAGCAGAAGACGGCATACGAGAT**CACTGT**GTGACTGGAGTTCAGACGT
P7 Index 6	CAAGCAGAAGACGGCATACGAGAT**ATTGGC**GTGACTGGAGTTCAGACGT
P7 Index 7	CAAGCAGAAGACGGCATACGAGAT**GATCTG**GTGACTGGAGTTCAGACGT
P7 Index 8	CAAGCAGAAGACGGCATACGAGAT**TCAAGT**GTGACTGGAGTTCAGACGT
P7 Index 9	CAAGCAGAAGACGGCATACGAGAT**CTGATC**GTGACTGGAGTTCAGACGT
P7 Index 10	CAAGCAGAAGACGGCATACGAGAT**AAGCTA**GTGACTGGAGTTCAGACGT
P7 Index 11	CAAGCAGAAGACGGCATACGAGAT**GTAGCC**GTGACTGGAGTTCAGACGT
P7 Index 12	CAAGCAGAAGACGGCATACGAGAT**TACAAG**GTGACTGGAGTTCAGACGT
U6-Illumina-seq2	TCTTCCGATCTCTTGTGGAAAGGACGAAACACCG

Supplementary Table 6. CRISPR screen raw sgRNA counts.

See separate Text file 'Mellor et al Table S6_sgRNA_count.txt'.

Output of the MAGeCK `count` command aligning Illumina sequencing reads to the sgRNA sequences within the Human Improved Genome-wide Knockout CRISPR library, following CRISPR/Cas9 screens in WT and *primpol* TK6 cells (Figure 1).

Supplementary Table 7. CRISPR screen untreated summary.

See separate Excel sheet 'Mellor et al Table S7_untreated_summary.txt'.

Output of the MAGeCK `test` command comparing sgRNA abundance between the sequencing libraries produced following a CRISPR/Cas9 knockout screen in WT versus *primpol* TK6 cells in untreated conditions (Figure 1B).

Supplementary Table 8. CRISPR screen cisplatin treated summary.

See separate Excel sheet 'Mellor et al Table S8_cddp_treated_summary.txt'.

Output of the MAGeCK `test` command comparing sgRNA abundance between the sequencing libraries produced following a CRISPR/Cas9 knockout screen in WT versus *primpol* TK6 cells challenged with 7 days continuous 0.25 µM cisplatin treatment (Figure 1C).

Supplementary Tables 6 – 8 can also be found on Zenodo as Excel files: doi: 10.5281/zenodo.8232516

Supplementary Materials and Methods

Amplification of the genome-wide CRISPR/Cas9 knockout library

110 ng of the Human Improved Genome-wide Knockout CRISPR library (7) obtained from Addgene (#67989, kind gift of Kosuke Yusa) was added to 10-beta electrocompetent *E. coli* (NEB) on ice. Electroporation was performed using chilled 1 mm electroporation cuvettes (Flowgen) and a GenePulser electroporator (Bio-Rad) using the following conditions: 2000 V, 200 Ω, 25 µF. 10-beta/Stable Outgrowth Medium (NEB), pre-warmed to 37˚C, was immediately added following transformation, gently mixing. The cultures were pooled and allowed to recover at 37ºC for 1 hour, 220 rpm shaking. 250 µL of the pooled culture and negative control transformation was plated at 1 in 10 dilutions up to 1 in 10^6 on 10 cm petri dishes containing 100 µg / mL ampicillin (Formedium) LB agar to assess coverage. The remainder was plated across eight 25 cm x 25 cm assay plates containing 100 µg / mL ampicillin (Formedium) LB agar. Plates were incubated for 15 hours at 37˚C. LB broth with 100 µg / mL ampicillin (Formedium) was added to each plate, the bacterial colonies scraped off and the resulting culture pooled. The culture was grown at 37˚C for 1 hour, 220 rpm shaking. 20% of the culture volume was used to prepare a glycerol stock. The remaining culture was centrifuged at 3200 g for 15 minutes to pellet bacteria. The pellet was weighed, and a Plasmid Plus Maxiprep (Qiagen) used to extract plasmid DNA. Isopropanol precipitation was performed to prepare a 2 µg / µL stock.

Generation of knockout cell lines

The Neon Transfection System (Invitrogen) was used to transfect TK6 cells. 30 µg plasmid DNA was added to 100 µL Buffer R. 4 x 10^6 TK6 cells were washed once using PBS and resuspended in the Buffer R/plasmid mix. The mixture was collected using a Neon 100 µL tip and transfected using a 1500 V, 20 ms single pulse with electroporation buffer E2. sgRNA sequences were designed using the CRISPOR online tool (8).

TK6 TSCER2 *primpol* cells utilised for the CRISPR/Cas9 screen were generated by transfection of TK6 TSCER2 cells with the construct pX458 sg*PRIMPOL* e5 (Supplementary Table 3). 2 days post-transfection GFP-expressing, propidium iodide-negative single cells were sorted into 96 well plates. Single cell-derived clones were probed for biallelic indels by Sanger sequencing, amplifying the targeted region of the genome using the primers *PRIMPOL* indel

F/R (Supplementary Table 5). Loss of PRIMPOL expression was confirmed by Western blot using the anti-CCDC111/PRIMPOL antibody listed in Supplementary Table 1.

TK6 TSCE5 *rev1* cells were generated by transfecting TK6 TSCE5 cells with the constructs pX458 sg*REV1* U1/U2/D1/D2 (Supplementary Table 3). 2 days post-transfection, GFP-expressing, propidium iodide-negative single cells were sorted into 96 well plates. Single cell-derived clones were probed for large genomic deletions using primers indicated in Supplementary Table 5. Loss of REV1 expression was confirmed by Western blotting (Supplementary Table 1).

Knockout cell lines (*bod1l1*, *edc4*, *paxip1*, *polh*, *polk*, *poli*, *hltf*, *smarcal1*, *rad18* and *primpol*) were generated in the TK6 TSCE5 background using CRISPR/Cas9-assisted introduction of targeting constructs (Supplementary Table 3). 4.5 µg of each pX458 construct and 6 µg of each targeting construct were used per transfection. 48 hours post-transfection, 1000 cells-per-well were plated in 96 well plates. Selection of cells which integrated the two targeting constructs used 2 mg / mL G418 (Formedium), 0.5 µg / mL puromycin dihydrochloride (Sigma-Aldrich), 7.5 µg / mL blasticidin S-hydrochloride (Sigma-Aldrich), 0.625 mg / mL hygromycin B (Sigma-Aldrich) and/or 0.5 mg / mL L-histidinol dihydrochloride (Sigma-Aldrich) as appropriate. Targeting was confirmed by genotyping PCR reactions (Supplementary Table 5). Loss of protein expression was confirmed by Western blotting (Supplementary Table 1).

Generation of cells expressing Cas9

Cas9-expressing cells were generated by transfection with the constructs pBS *AAVS1*::Cas9-YFP-BSD and pX330 sg*AAVS1* (Supplementary Table 3). 48 hours post-transfection, 1000 cells-per-well were plated in 96 well plates with 7.5 µg / mL blasticidin S-hydrochloride (Sigma-Aldrich). Clones were isolated and heterozygous insertions into the *AAVS1* locus confirmed by genotyping PCR (Supplementary Table 5). Stable expression of YFP was confirmed by flow cytometry using a BD LSRFortessa Cell Analyser (BD Bioscience)

Cas9 activity was assayed by loss of cell surface expression of MHC Class I following transduction with lentivirus delivering an equimolar pool of five sgRNAs targeting *B2M* (pKLV2 U6-g*B2M*1-5) or the corresponding empty vector (Supplementary Table 3) with a target MOI of 0.3. 7 days post-transduction, MHC class I knockdown was assessed by staining with anti-HLA-ABC, APC (Invitrogen W6, 32), diluted 1 in 500 in PBS, incubating for 15

minutes at room temperature, followed by washing once in PBS. Flow cytometry used a BD LSRFortessa Cell Analyser (BD Bioscience).

Generation of pcnaK164R cells

*pcna*K164R cells were generated through targeted CRISPR/Cas9-assisted genome editing through homology-directed repair. Transfection utilised the Neon Transfection System (Invitrogen) with Neon 10 µL tips. TK6 TSCE5 cells were co-transfected with a single-stranded Alt-R HDR donor oligonucleotide (Supplementary Table 3) and electroporation enhancer alongside tracrRNA, crRNA (TTTCACTCCGTCTTTTGCAC) and AltR S.p Cas9 nuclease combined to form Cas9 ribonucleoprotein (all IDT), using the manufacturer's recommended conditions. 2 days post-transfection, cells were plated in 96 well plates to isolate single cell-derived clones by limiting dilution. To genotype, the genomic region containing the targeted modification was amplified by PCR using the primers *PCNA* e5 F and *PCNA* e5 R (Supplementary Table 5). Initial screening utilised a new ApaLI site introduced during the editing process. Following this, the desired genome editing events were confirmed through Sanger sequencing of the bulk PCR product using the primer PCNA K164R seq (Supplementary Table 5).

Preparation of viral vectors and transduction of TK6 cells

To package the Human Improved Genome-wide Knockout CRISPR library into lentivirus, 6 well plates containing 80% confluent HEK293ET cells in 2.5 mL fresh medium were prepared. For each well, 1.2 µg pSPAX2 (Addgene #12260, kind gift of Didier Trono), 0.25 µg pMD2.G (Addgene #12259, kind gift of Didier Trono) and 0.5 µg of the amplified genome-wide library were added to 250 µL Opti-MEM Reduced Serum Medium (Gibco). 6 µL TransIT lentivirus system (Mirus) was added, the mixture incubated at room temperature for 30 minutes, before adding to each well of HEK293ET cells. Medium was discarded after 24 hours and replaced with fresh HEK 293ET medium. After 48 and 72 hours, the medium was collected and pooled (adding 2.5 mL fresh medium at 48 hours). The pooled medium was centrifuged twice (420 g, 5 minutes), each time collecting the supernatant, followed by aliquoting the virus-containing supernatant and storing at -80°C until use. The lentivirus was titrated by transduction into TK6 cells, assessing transduction efficiency from tagBFP expression using a BD LSRFortessa Cell Analyser (BD Bioscience). To package complementation constructs into gammaretrovirus, an identical protocol to that used for lentivirus packaging was used except that the plasmids co-

transfected are 0.875 μg pMD-OGP, 0.375 μg pMD-VSV-G and 1.25 μg of the indicated M6P plasmid.

For transduction, 6 x 10^6 cells TK6 cells were seeded in 2.5 mL medium with 4 μg / mL Polybrene (Sigma) in 6 well plates. Either lentivirus or gammaretrovirus preparation was added, and cells were centrifuged at 650 g (90 minutes, 37°C). Cells were incubated at 37°C, 5% CO_2 for 1 hour to allow recovery. Following this, cells were resuspended and added to the appropriate volume of TK6 medium for culture.

Complementation

RAD18 complementation was performed using the construct BML3 YFP-hRAD18 (Supplementary Table 3). POLη complementation was performed using the construct BML3 YFP-hPolη (Supplementary Table X). In both cases, stable non-targeted transfection using the Neon Transection System (Invitrogen) was used to introduce the complementation constructs. Single cell-derived clones were isolated by plating 500 cells-per-well into 96 well plates with 2 mg / mL G418 (Formedium). Stable expression of the YFP-tagged proteins was confirmed by flow cytometry using a BD LSRFortessa Cell Analyser (BD Bioscience) and Western blot (Supplementary Table 1)

PRIMPOL and REV1 complementation was performed using the M6P constructs (Supplementary Table 3) packaged into gammaretroviral vectors as described below. Cells were transduced as described below to give a target MOI of 0.3. The transduced cells were selected as a pooled population with either 2 mg / mL G418 (Formedium) or 7.5 μg / mL blasticidin S-hydrochloride (Sigma-Aldrich) for 7 days. Expression of the YFP/GFP-tagged proteins was confirmed by flow cytometry using a BD LSRFortessa Cell Analyser (BD Bioscience) and Western blot (Supplementary Table 1).

DNA damaging agents

For UV-C irradiation, 1 million cells / mL were resuspended in PBS, and 1 mL transferred to wells of a 6 well plate. UV-C irradiation was performed using a custom-built shuttered cabinet (peak output: 265 nm). Stabilised bulb output was measured with a calibrated UV-C meter (UVP). Following irradiation, 4 mL fresh medium was added, and cells were incubated for 1 hour at 37°C in 5% CO_2 to allow recovery, before resuspension and transfer to a larger volume of medium. Cisplatin (cis-Diammineplatinum(II) dichloride) was sourced from Sigma-Aldrich.

Fresh 1 mM stocks were produced prior to use by dissolving in 0.9% (w/v) NaCl. Methane methylsulfonate (Sigma-Aldrich) was dissolved 1 in 1000 (v/v) in TK6 cell culture medium prior to use – doses were expressed in parts-per-million (ppm) according to the dilution used. Mitomycin C (Sigma Aldrich) was dissolved in H_2O to prepare a 0.2 mg / mL stock solution and aliquots stored at -20°C. 4-nitroquinolone 1-oxide was dissolved in DMSO to give a 50 mM stock solution and aliquots stored at -80°C.

Western blots

For validating KO cell lines and assessing PRIMPOL upregulation following cisplatin treatment, protein was extracted by direct lysis of 1 million cells in 100 µL 2X Laemlli buffer supplemented with *β-Mercaptoethanol* (BioRad), incubating at 95°C for 10 minutes followed by sonication. To assess PCNA ubiquitination, 1 million cells were collected, washed once in PBS and flash frozen in liquid nitrogen, storing at -80°C until protein extraction. Protein extraction was performed using RIPA buffer (Cell Signalling), supplemented with 10 mM N-ethylmalemide (Sigma-Aldrich) in addition to Halt Protease Inhibitor Cocktail (Thermo Scientific) and Benzonase Nuclease (Sigma-Aldrich).

Antibodies used for Western blots are listed in Supplementary Table 5. Polyacrylamide gel electrophoresis was performed using NuPAGE 4-12% BisTris Precast Polyacrylamide Gels (Invitrogen) alongside PageRuler or PageRuler Plus Prestained Protein Ladder (Invitrogen). The gel was electrophoresed at 50V for 10 minutes, and then subsequently at 125 V until the dye front reached the base of the gel. MOPS SDS (50 mM MOPS, 50 mM Tris, 1 mM EDTA, 0.1% SDS, pH 7.7) was used as a running buffer. Protein transfer was performed either using the iBlot 2 system (Invitrogen) with iBlot2 Transfer Stacks, Nitrocellulose (Invitrogen), transferring at 25 V for 7 minutes, or through standard wet-transfer protocols using Amershan Protran 0.45 µm Nitrocellulose Blotting Membrane (Amershan), as indicated in Supplementary Table 5. Post-transfer the membrane was dried for 1 hour, followed by Ponceau S (Cell Signalling Technology) staining for around 2 minutes (room temperature, rotating) until bands appeared, washing twice in TBS (150 mM NaCl, Tris HCl pH 7.4 10 mM) before visualisation. The membrane was blocked in 5% milk (Marvel) dissolved in TBS-T (TBS, 0.1% Tween 20) for 1 hour, rotating at room temperature. The primary antibodies were diluted in 5% milk in TBS-T, rotating overnight at 4°C. The membranes were washed 3 x 10 minutes in TBS-T. The membranes were rotated at room temperature for 1 hour in Goat Anti-Mouse/Rabbit Immunoglobulins/HRP secondary antibodies (Aglient Dako) diluted 1 in 10000 in 5% milk in

TBS-T. The membranes were washed 3 x 10 minutes in TBS-T, followed by a 1-minute wash in TBS. The membrane was transferred to a clean section of plastic. 1 mL per 10 cm^2 membrane Radiance Plus Femtogram HRP Substrate (Azure Biosystems) was added, diluting as required when the signal was strong, incubating for 5 minutes. The membrane was transferred to a cassette, and the blot revealed in a dark room using X-ray film, processing using a film developer.

Supplementary References

1. Randow,F. and Sale,J.E. (2006) Retroviral transduction of DT40. *Subcell Biochem* **40**, 383-386.
2. Szüts,D., Simpson,L.J., Kabani,S., Yamazoe,M. and Sale,J.E. (2006) Role for RAD18 in homologous recombination in DT40 cells. *Mol Cell Biol* **26**, 8032-8041.
3. Ross,A.L., Simpson,L.J. and Sale,J.E. (2005) Vertebrate DNA damage tolerance requires the C-terminus but not BRCT or transferase domains of REV1. *Nucleic Acids Res* **33**, 1280-1289.
4. Arakawa,H., Lodygin,D. and Buerstedde,J.M. (2001) Mutant loxP vectors for selectable marker recycle and conditional knock-outs. *BMC Biotechnol* **1**, 7.
5. Schiavone,D., Jozwiakowski,S.K., Romanello,M., Guilbaud,G., Guilliam,T.A., Bailey,L.J., Sale,J.E. and Doherty,A.J. (2016) PrimPol Is Required for Replicative Tolerance of G Quadruplexes in Vertebrate Cells. *Mol Cell* **61**, 161-169.
6. Edmunds,C.E., Simpson,L.J. and Sale,J.E. (2008) PCNA ubiquitination and REV1 define temporally distinct mechanisms for controlling translesion synthesis in the avian cell line DT40. *Mol Cell* **30**, 519-529.
7. Tzelepis,K., Koike-Yusa,H., De Braekeleer,E., Li,Y., Metzakopian,E., Dovey,O.M., Mupo,A., Grinkevich,V., Li,M., Mazan,M., Gozdecka,M., Ohnishi,S., Cooper,J., Patel,M., McKerrell,T., Chen,B., Domingues,A.F., Gallipoli,P., Teichmann,S., Ponstingl,H., McDermott,U., Saez-Rodriguez,J., Huntly,B.J.P., Iorio,F., Pina,C., Vassiliou,G.S. and Yusa,K. (2016) A CRISPR Dropout Screen Identifies Genetic Vulnerabilities and Therapeutic Targets in Acute Myeloid Leukemia. *Cell Rep* **17**, 1193-1205.
8. Concordet,J.P. and Haeussler,M. (2018) CRISPOR: intuitive guide selection for CRISPR/Cas9 genome editing experiments and screens. *Nucleic Acids Res* **46**, W242-W245.
